# Increased p53 expression induced by APR-246 reprograms tumor-associated macrophages to augment immune checkpoint blockade

**DOI:** 10.1172/JCI148141

**Published:** 2022-09-15

**Authors:** Arnab Ghosh, Judith Michels, Riccardo Mezzadra, Divya Venkatesh, Lauren Dong, Ricardo Gomez, Fadi Samaan, Yu-Jui Ho, Luis Felipe Campesato, Levi Mangarin, John Fak, Nathan Suek, Aliya Holland, Cailian Liu, Mohsen Abu-Akeel, Yonina Bykov, Hong Zhong, Kelly Fitzgerald, Sadna Budhu, Andrew Chow, Roberta Zappasodi, Katherine S. Panageas, Olivier de Henau, Marcus Ruscetti, Scott W. Lowe, Taha Merghoub, Jedd D. Wolchok

**Affiliations:** 1 Swim Across America and Ludwig Collaborative Laboratory, Immunology Program, Parker Institute for Cancer Immunotherapy,; 2 Immuno-Oncology Service, Human Oncology and Pathogenesis Program,; 3 Department of Medicine, and; 4 Department of Cancer Biology and Genetics, Sloan Kettering Institute, Memorial Sloan Kettering Cancer Center, New York, New York, USA.; 5 Rockefeller University, New York, New York, USA.; 6 Department of Epidemiology and Biostatistics, Memorial Sloan Kettering Cancer Center, New York, New York, USA.; 7 Howard Hughes Medical Institute, Chevy Chase, Maryland, USA.

**Keywords:** Oncology, Therapeutics, Cancer immunotherapy, Immunotherapy, p53

## Abstract

In addition to playing a major role in tumor cell biology, p53 generates a microenvironment that promotes antitumor immune surveillance via tumor-associated macrophages. We examined whether increasing p53 signaling in the tumor microenvironment influences antitumor T cell immunity. Our findings indicate that increased p53 signaling induced either pharmacologically with APR-246 (eprenetapopt) or in p53-overexpressing transgenic mice can disinhibit antitumor T cell immunity and augment the efficacy of immune checkpoint blockade. We demonstrated that increased p53 expression in tumor-associated macrophages induces canonical p53-associated functions such as senescence and activation of a p53-dependent senescence-associated secretory phenotype. This was linked with decreased expression of proteins associated with M2 polarization by tumor-associated macrophages. Our preclinical data led to the development of a clinical trial in patients with solid tumors combining APR-246 with pembrolizumab. Biospecimens from select patients participating in this ongoing trial showed that there was a suppression of M2-polarized myeloid cells and increase in T cell proliferation with therapy in those who responded to the therapy. Our findings, based on both genetic and a small molecule–based pharmacological approach, suggest that increasing p53 expression in tumor-associated macrophages reprograms the tumor microenvironment to augment the response to immune checkpoint blockade.

## Introduction

A major hurdle in the successful application of immunotherapies is poor infiltration of functional antitumor T cells in the immunosuppressive tumor microenvironment (TME) (1, 2). Infiltrating myeloid cells that include tumor-associated macrophages (TAMs) are a heterogeneous but key constituent of the TME (3). These cells are known to prevent infiltration and suppress efficacy of T cells, thereby decreasing antitumor immunity (4, 5). TAMs have diverse phenotypes and can be roughly divided into M1 and M2 subtypes corresponding to Th1 and Th2 polarization: M2 TAMs induced in culture conditions under the influence of IL-4 and M-CSF and M1 TAMs that are typically induced under the influence of IFN-γ (6). M2-like TAMs, particularly enriched in tumors, express cytokines such as IL-10 and prototypic markers such as arginase 1 (Arg-1), indolamine-2,3-dioxygenase (IDO), and CD206 (mannose receptor C, MRC) and suppress T cell activity through production of immunosuppressive metabolites (4, 7). M1 TAMs, on the other hand, express inflammatory cytokines such as IL-12, IL-6, and markers such as major histocompatibility complex class II (MHC-II) and inducible nitric oxide synthase (NOS2), thereby facilitating T cell effector functions (8, 9). Targeting intracellular signaling pathways in TAMs can reprogram the TME and combination strategies using drugs that target TAMs with immune checkpoint blockade (ICB) hold the potential to improve outcomes of cancer immunotherapy independently of the intrinsic features of the tumors themselves.

p53 (encoded by *TP53* in humans and *Trp53* in mice) can exert immunomodulatory effects on the TME; for example, p53-mediated induction of cellular senescence triggers a secretory program known as senescence-associated secretory phenotype (SASP) that modulates immune responses in the TME (10). Once initiated, SASP reinforces the senescence program and influences immune surveillance, forming a critical interphase between tumor cells and the innate immune cells such as TAMs. Targeted cancer therapies can act on pathways associated with senescence in tumor cells and induce SASP (11, 12). However, SASP is complex and heterogeneous, such that the inflammatory effects of SASP can also be protumorigenic and detrimental to antitumor immune responses (13). Recent data suggest that induction of a partial SASP that is enriched in targets of the p53 pathway is more permissive for antitumor immunity (14). While the non–cell autonomous effects of SASP on myeloid cells in the TME have been described, the direct effects of senescence-triggering therapies on TAMs are less well understood.

Although the p53 pathway remains one of the most frequently altered in cancer, drugs directly targeting p53 have been difficult to develop (15). However, the landscape of p53-targeting drugs has evolved and diverse modalities of drugs activating p53-regulatory proteins have recently entered clinical trials. APR-246 (eprenetapopt) is a small molecule that works via a distinct mechanism, independent of negative regulators of p53, whereby it structurally stabilizes the p53 protein by reversibly binding to its core domain through alkylating thiol groups and shifting the equilibrium toward a folded, active p53 conformation (16). APR-246 is currently being evaluated in clinical trials to explore therapeutic effects in acute myeloid leukemia and myelodysplastic syndrome with p53 mutations, where it can bind to mutant p53 in leukemic cells to restore its activity (17, 18). While the effects of APR-246 on mutant p53 in tumor cells are well characterized, its effects on enhancing wild-type (WT) p53, especially in immune cells of the TME, are not as well studied. Therefore, we sought to explore the role of APR-246 in cells with WT p53 and here demonstrate that increased p53 in immune cells of the TME is associated with a decrease in immunosuppressive TAMs that reprogram the TME. We also show that there is an increased antitumor activity when methods to enhance p53 are combined with ICB in preclinical mouse models of melanoma and in patients. We further show that treatment with APR-246 or the analogous use of transgenic mice with increased genetic dosage of p53 intrinsically reprograms macrophages to overcome resistance to ICB and thus improves therapeutic efficacy.

## Results

### APR-246 therapy enhances response to ICB.

**Figure 1:**
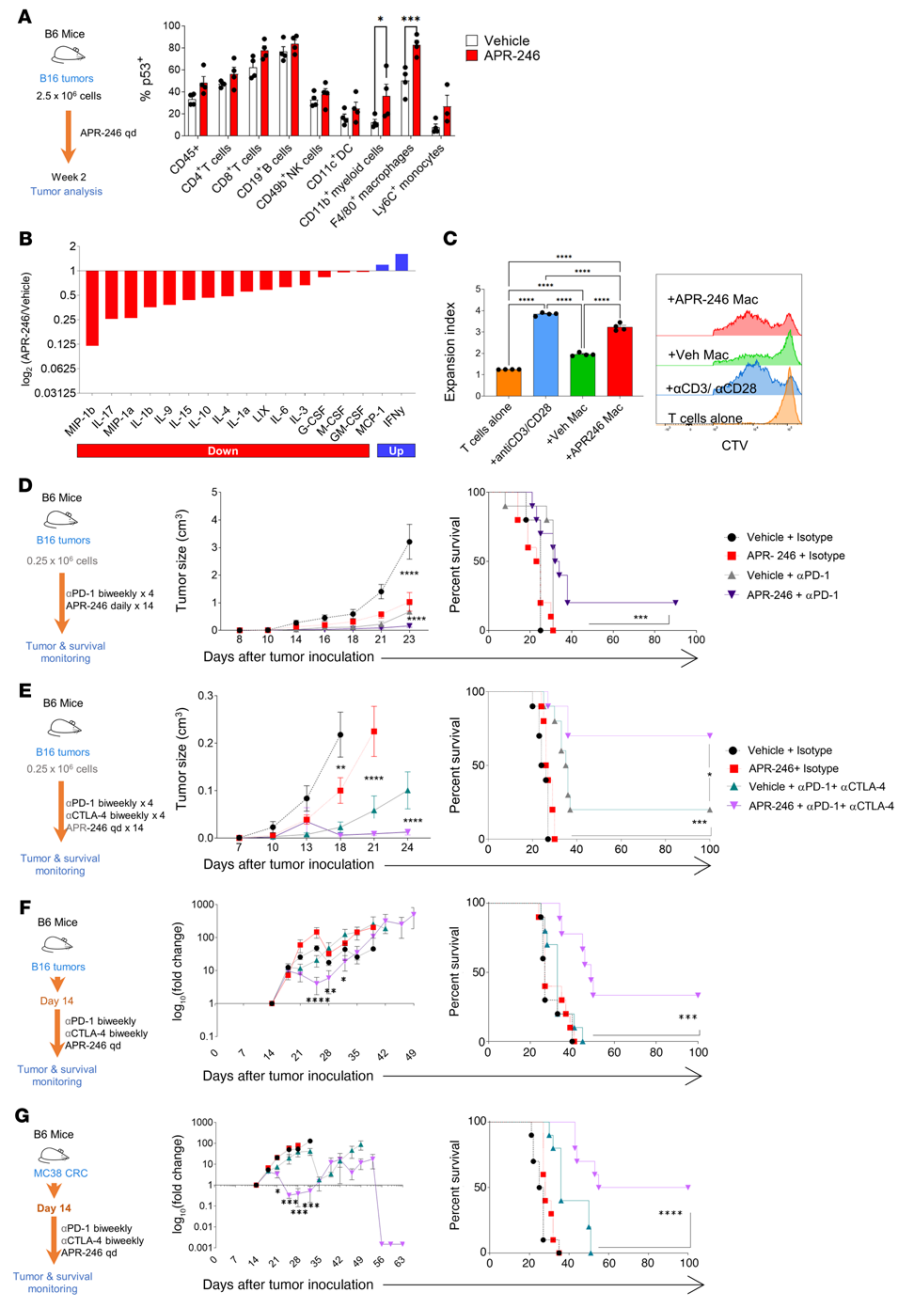
APR-246 augments the effects of PD-1 blockade in mouse models. (**A**) Schematic showing analysis of the TME of B16 tumors in APR-246– versus vehicle-treated mice. Tumors were harvested on day 13. Flow cytometry was performed and frequency of p53^+^ events in gated immune subsets are depicted (*n* = 3–5/group, mean ± SEM shown). (**B**) Murine cytokine array for tumor lysates. Bars represent the ratio of the mean intensity of 3 biologically independent experiments with 3 technical replicates each (log_2_). Factors relatively increased (blue) or decreased (red) on tumors from mice treated with APR-246 versus PBS (vehicle) are shown (*n* = 9/group, performed in duplicate; the ratio of means shown). (**C**) Sorted CD11b^+^F4/80^+^ TAMs were cocultured with CTV-labeled CD8^+^ T cells that were magnetically sorted from non–tumor-bearing B6 mice as well as anti-CD3/anti-CD28–coated activating beads. CTV dilution was detected by flow cytometry and expansion index calculated by FlowJo. Plot is representative of 3 independent experiments. (**D**) Schematic of treatment, tumor growth curves, and Kaplan-Meier survival of B6 mice with B16 tumors when treated with APR-246 versus vehicle with and without anti–PD-1 antibody in B6 mice with B16 tumors (*n* = 10/group, representing 3 independent experiments). (**E**) Schematic of treatment, tumor growth curves, and Kaplan-Meier survival of B6 mice with B16 tumors when treated with APR-246 versus vehicle, with and without anti–PD-1 and anti–CTLA-4 (*n* = 10/group, representing 3 independent experiments). (**F**) Schematic of treatment, mean fold change (vs. pretreatment), and Kaplan-Meier survival in tumor growth of B16 tumors in B6 mice treated with APR-246 versus vehicle, with and without anti–PD-1 and anti–CTLA-4 (*n* = 10/group, representing 2 independent experiments). (**G**) Schematic of treatment, mean fold change (vs. pretreatment) in tumor growth, and Kaplan-Meier survival of MC38 colorectal carcinoma (CRC) tumors in B6 mice treated with APR-246 versus vehicle, with and without anti–PD-1 and anti–CTLA-4 in B6 mice with MC38 CRC tumors (*n* = 10/group, representing 2 independent experiments). *P < 0.05; **P < 0.01; ***P < 0.001; ****P < 0.0001 by 2-way ANOVA with multiple *t* tests corrected with Bonferroni’s method (**A** and **D**–**G** [tumor growth]), 1-way ANOVA with multiple *t* tests corrected with Bonferroni’s method (**C**), or Kaplan-Meier with results ranked by Mantel-Cox log-rank test (**D**–**G** [survival patterns]).

In order to study the effect of enhanced p53 activity on the TME independently of its effect on p53-mutant tumor cells, we implanted B16 melanoma cells that have a functional *Trp53* gene in C57BL/6J mice and treated these mice daily with APR-246 or vehicle control (PBS) starting 1 week after implantation (Figure 1A). Analyses of the immune cells of the TME by flow cytometry revealed increased expression of p53 in CD11b^+^ myeloid cells and F4/80^+^ TAMs in mice treated with APR-246 (Figure 1A). Thus, treatment with APR-246 results in increased WT p53 levels in the infiltrating immune myeloid cells of the TME.

We next analyzed the effect of APR-246 treatment on the composition of chemokines and cytokines secreted in the TME by performing a multiplexed array on tumor lysates from vehicle- and APR-246–treated mice (Figure 1B). These analyses revealed relative decreases in chemokines and cytokines such as MIP-1, IL-1, IL-10, and IL-4 that are associated with M2 polarization of TAMs and a relative increase in IFN-γ, which is associated with M1 polarization.

To determine how treatment with APR-246 affects T cell functions through myeloid/macrophage cells in the TME, we sorted TAMs (CD45^+^CD11b^+^TCRβ^–^F4/80^+^) from vehicle- and APR-246–treated mice. We used these cells in a coculture suppression assay with CellTrace Violet–labeled (CTV-labeled) WT CD8^+^ T cells from non–tumor-bearing mice (Figure 1C). CD8^+^ T cells cocultured with TAMs derived from APR-246–treated mice showed a higher expansion index compared with TAMs from vehicle-treated mice, suggesting that TAMs in the APR-246–treated TME promote T cell proliferation. Taken together, these results suggest that APR-246 could reprogram TAMs to promote an antitumoral T cell response.

Given the observed suppression of M2-associated chemokines and cytokines, and T cell proliferative effects of TAMs associated with APR-246 treatment, we investigated the efficacy of combining ICB with APR-246 in multiple immunocompetent murine tumor models. Monotherapy with either anti–programed death 1 (anti–PD-1) antibody (RMP1-14) or APR-246 led to minimal tumor control in the B16 melanoma model (Figure 1D and Supplemental Figure 1A; supplemental material available online with this article; https://doi.org/10.1172/JCI148141DS1). However, the combination of anti–PD-1 with APR-246 led to a significant delay in tumor progression (*P* < 0.001) and improved survival of WT B16-bearing mice compared with either monotherapy (*P* < 0.01). Improved control of tumor and survival with the combination of anti–PD-1 and APR-246 was also seen in an MC38 colorectal carcinoma model (Supplemental Figure 1B) and TC1, an HPV tumor model (Supplemental Figure 1C). Tumor control was lost in nude mice that lack T cells, thus suggesting that T cells are required for the efficacy of APR-246 and anti–PD-1 combination therapy (Supplemental Figure 1D).

To further enhance the effect of combining APR-246 with ICB, we tested dual ICB using a combination of antibodies that block PD-1 and cytotoxic T lymphocyte–associated antigen 4 (CTLA-4), a strategy that has been shown to elicit a high response rate in patients with melanoma (19, 20). We therefore combined APR-246 with anti–PD-1 (RMP1-14) and anti–CTLA-4 (9D9) in B16-melanoma-bearing mice and found a significantly longer survival compared with dual ICB alone (*P* < 0.001) (Figure 1E and Supplemental Figure 1E). We next investigated whether the combination of APR-246 with dual ICB could decrease the size of established tumors by delaying the initiation of therapy and continuing therapy through the lifespan of the experimental mice (Figure 1, F and G). B16 melanoma tumors treated with dual ICB had transient decreases in progression in 20% of the mice. In contrast, mice treated with APR-246 plus dual ICB had a significantly larger decrease, with decreased tumor size in 50% of the mice, durable decreases in 30% of the mice, and an improved overall survival (Figure 1F and Supplemental Figure 2A). An augmented response to APR-246 plus dual ICB was also seen in the established MC38 colorectal adenocarcinoma (Figure 1G and Supplemental Figure 2B).

**Figure 2:**
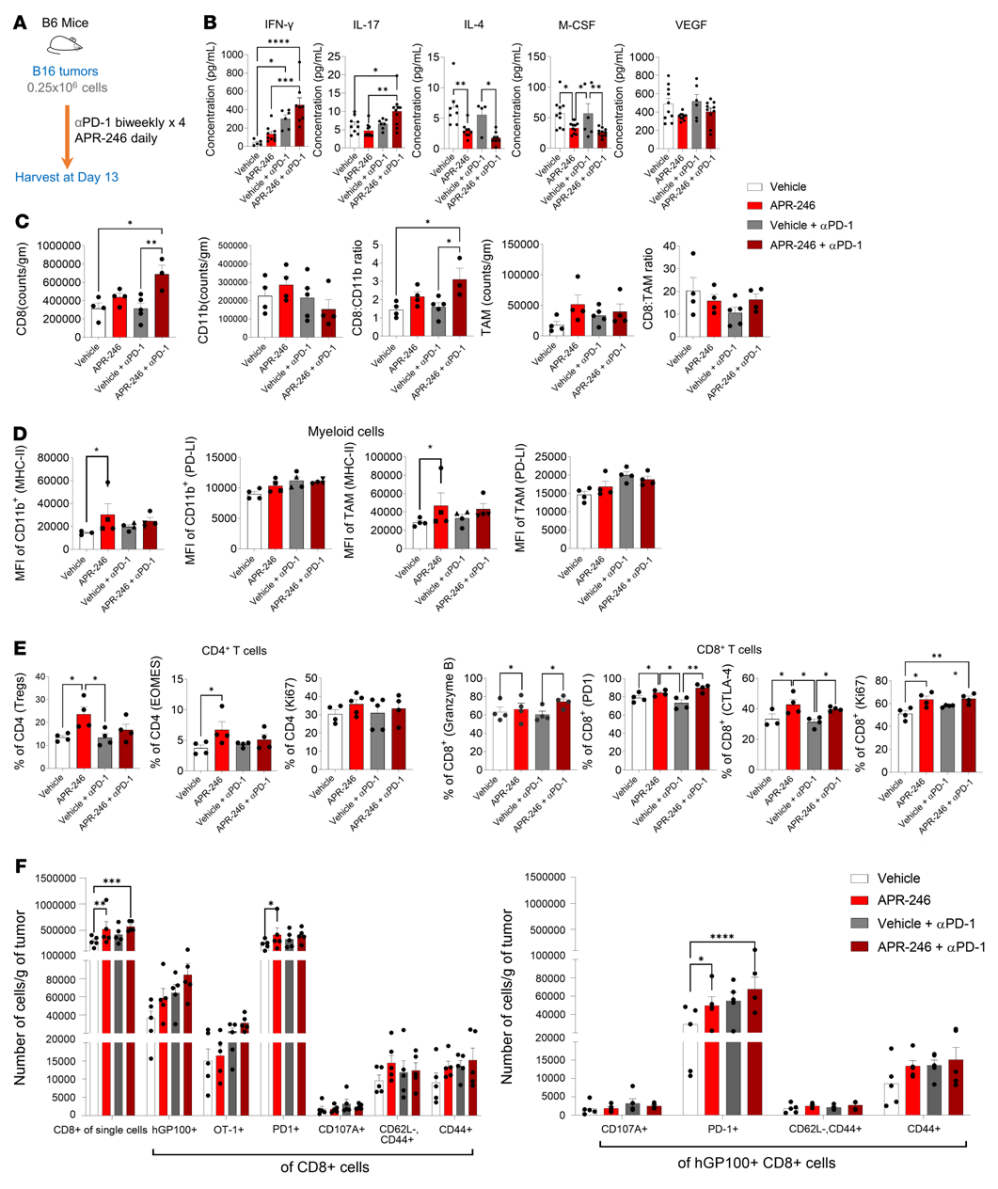
APR-246 reprograms the immune tumor microenvironment. (**A**) Schematic of treatment of B6 mice harboring B16 tumors with APR246 and vehicle (control). (**B**) Murine cytokine array performed on tumor lysates (*n* = 4–5/group, performed in duplicate; mean ± SEM shown). (**C**–**F**) Multicolor flow cytometry analyses of live CD45^+^ gated cells of the TME (*n* = 4–5/group, mean ± SEM shown). (**C**) Flow cytometry analyses enumerating CD8^+^ T cells, CD11b^+^ myeloid cells, and CD11b^+^F4/80^+^ TAMs. (**D**) Phenotypic characterization of CD11b^+^ myeloid cells and CD11b^+^F4/80^+^ TAMs, and (**E**) CD4^+^ and CD8^+^ T cells. (**F**) Number of putative melanoma-specific hGP100TCR^+^ T cells (vs. control SIINFEKL-specific OT-1 TCR^+^ T cells [left panel] and their phenotype [right panel]) as present in the TME. *P < 0.05; **P < 0.01; ***P < 0.001; ****P < 0.0001 by 1-way ANOVA with multiple *t* tests corrected with Bonferroni’s method (**A**–**E**) or 2-way ANOVA with multiple *t* tests corrected with Bonferroni’s method (**F**).

We next performed in-depth analyses of the effect of APR-246 and concurrent use of PD-1 blockade on the TME of B16 melanoma (Figure 2A). The B16 TME had higher levels of T cell–potentiating cytokines such as IFN-γ, and lower levels of the T helper 2–associated (Th2-associated) IL-4 (Figure 2B) with the use of APR-246 and PD-1 blockade. IL-17, which was mildly decreased with APR-246 alone, was significantly increased when APR-246 was combined with PD-1 blockade, compared with either monotherapy alone. Concurrently, cytokines associated with chronic inflammation that can trigger T cell suppression, including M-CSF (Figure 2B), IL-10, IL-1β, IL-6, and MIP-1 (Supplemental Figure 3A) were decreased. MCP-1, which was mildly increased with APR-246 alone, was significantly decreased when APR-246 was combined with PD-1 blockade, compared with either monotherapy control.

In line with these cytokine levels, flow cytometry revealed a decrease in the overall frequency of CD4^+^ T cells and an increase in CD8^+^ T cells (Supplemental Figure 3B). We found a small increase in CD25^+^CD4^+^ T cells with APR-246 therapy (Supplemental Figure 3C). There was also a significant decrease in CD62L^+^CD8^+^ T cells among APR-246–treated groups, suggesting a decrease in the naive CD8^+^ T cell subset. Concurrently, we found an increase in CD44^+^CD8^+^ T cells, suggesting an increase in the memory CD8^+^ T cell subset. Importantly, we found a significant increase in the number of CD8^+^ T cells and the ratio of the number of CD8^+^ T cells per gram of tumor to the number of CD11b^+^ myeloid cells per gram of tumor with APR-246 combination therapy (Figure 2C). APR-246 therapy also induced higher expression of MHC-II on CD11b^+^ myeloid cells and F4/80^+^ TAMs (Figure 2D). Additionally, there were higher frequencies of MHC-II^+^ myeloid cells and lower frequencies of CD206^+^ cells, indicating a greater infiltration of MHC-II^+^ classically activated M1 TAMs (Supplemental Figure 3D). Further analyses of the T cells revealed that the CD4^+^ T cells from APR-246–treated mice showed a higher frequency of Foxp3^+^ T regulatory (Treg) cells and eomes without PD-1 blockade, but these differences were largely reversed with PD-1 blockade (Figure 2E and Supplemental Figure 3E). On the other hand, among the CD8^+^ T cells, we found increased cytotoxicity denoted by granzyme B, increased proliferation denoted by Ki67, and increased expression of targets of ICB therapy such as cells expressing PD-1, CTLA-4, and glucocorticoid-induced TNFR-related protein (Gitr) in the APR-246–treated group (Figure 2E and Supplemental Figure 3F). There was also an increased frequency of CD11c^+^ dendritic cells (DCs) in the APR-246–treated group (Supplemental Figure 3G).

The above-described data suggested that treatment with APR-246 induced a T cell–permissive TME and increased infiltration of CD8^+^ T cells, when assessed for the presence of B16-specific T cells in the TME of mice. The gp100 antigen (also known as premelanosome protein, PMEL) is an intracellular transmembrane glycoprotein enriched in melanosomes and B16 melanoma cells (21). Thus, T cells expressing gp100-specific T cell receptors (hGP100TCR^+^CD8^+^) are putative B16-targeting T cells. Compared with the vehicle-treated TME, the APR-246–treated TME had significantly more total CD8^+^ T cells but similar numbers of hGP100TCR^+^CD8^+^ T cells. However, the characterization of the CD8^+^ T cell phenotype shows that the APR-246 treatment induced higher numbers of hGP100TCR^+^CD8^+^ T cells expressing PD-1 in the TME (Figure 2F). Thus, treatment with APR-246 induces a T cell–permissive microenvironment with an increase in targets of ICB, leading to enhanced tumor control with combination immunotherapy.

### APR-246 therapy acts via increasing p53 in the tumor-associated myeloid/macrophage cells to enhance response to ICB.

APR-246 is known to mediate its effects by binding to thiol groups to stabilize and activate p53. Treatment with APR-246 can affect inflammation directly through enhanced p53 signaling in T cells and TAMs, or indirectly via non–cell autonomous effects of the drug on tumor or stromal cells of the TME or via off-target effects.

**Figure 3:**
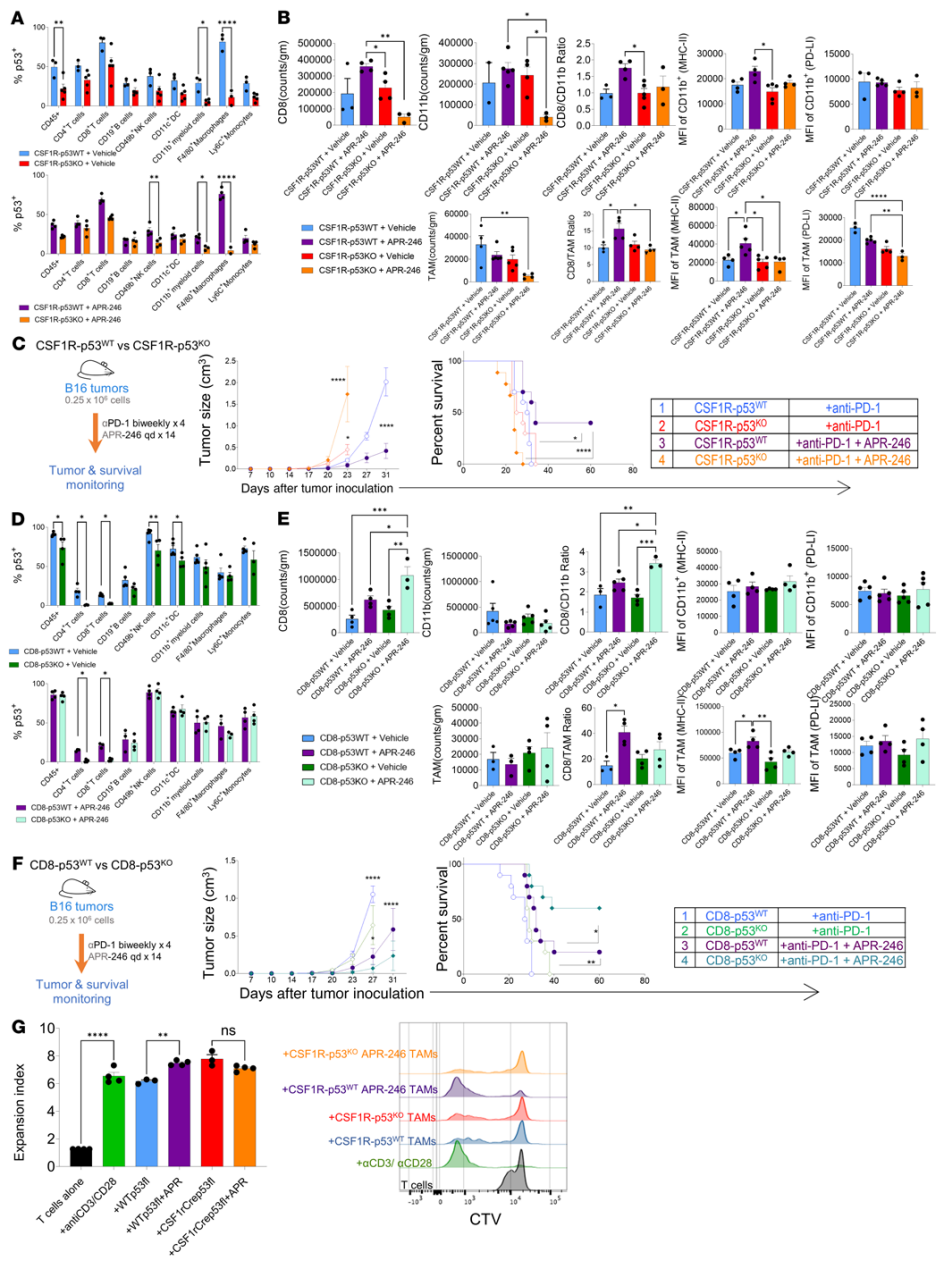
Loss of p53 in myeloid cells leads to loss of APR-246 combination therapy–mediated tumor control. (**A**–**C**) CSF1R-p53^KO^ versus CSF1R-p53^WT^ mice were treated with APR-246 starting on day 7 after B16 melanoma inoculation. Tumors were harvested on day 13 and flow cytometry was performed (*n* = 5/group, mean ± SEM shown). (**A**) Frequency of p53^+^ events in gated immune subsets, and (**B**) analyses enumerating CD8^+^ T cells, CD11b^+^ myeloid cells, and CD11b^+^F4/80^+^ TAMs. (**C**) Schematic of treatment, tumor growth curve, and Kaplan-Meier survival of CSF1R-p53^KO^ versus CSF1R-p53^WT^ with B16 tumors that were treated with APR-246 versus control with anti–PD-1 (each plot depicts 1 representative experiment, *n* = 9–10/group). (**D**–**F**) CD8-p53^KO^ versus CD8-p53^WT^ mice were treated with APR-246 starting on day 7 after B16 melanoma inoculation. Tumors were harvested on day 13 and flow cytometry was performed (*n* = 4–5/group, mean ± SEM shown). (**D**) Frequency of p53^+^ events in gated immune subsets, and (**E**) analyses enumerating CD8^+^ T cells, CD11b^+^ myeloid cells, and CD11b^+^F4/80^+^ TAMs. (**F**) Schematic of treatment, tumor growth curve, and Kaplan-Meier survival of CD8-p53^KO^ versus CD8-p53^WT^ mice harboring B16 tumors treated with APR-246 versus control with anti–PD-1 (each plot depicts 1 representative experiment, *n* = 9–10/group). (**G**) CD45^+^TCRβ^–^CD11b^+^F4/80^+^ TAMs were sorted from CSF1R-p53^KO^ versus CSF1R-p53^WT^ mice with B16 tumors that were treated with vehicle (PBS) or APR-246. These TAMs were cocultured with CTV-labeled CD8^+^ T cells magnetically sorted from non–tumor-bearing B6 mice as well as anti-CD3/anti-CD28–coated activating beads. CTV dilution was detected by flow cytometry and expansion index calculated by FlowJo. Plot is representative of 3 independent experiments. *P < 0.05; **P < 0.01; ***P < 0.001; ****P < 0.0001 by 2-way ANOVA with multiple *t* tests corrected with Bonferroni’s method (**A**, **C** [tumor growth], **D**, and **F** [tumor growth]), 1-way ANOVA with multiple *t* tests corrected with Bonferroni’s method (**B**, **E**, and **G**), or Kaplan-Meier with results ranked by Mantel-Cox log-rank test (**C** and **F** [survival patterns]).

To study the effects of APR-246 mediated through p53 in the myeloid components of the TME, we generated a tissue-specific knockout of p53 in CSF1R^+^ myeloid cells (CSF1R-p53^KO^; CSF1R^cre^ × p53^fl^ crossbreeding). Non–Cre-expressing littermates were used as control (CSF1R-p53^WT^). This conditional knockout causes loss of p53 in myeloid cells and thus p53 in CD11b^+^ myeloid cells, and F4/80^+^ TAMs cannot be augmented by APR-246, while p53 in the T cells of these mice remains intact (Figure 3A). The TME from CSF1R-p53^KO^ mice had a decreased CD8^+^ T cell infiltration, decreased CD8^+^ T cell/CD11b^+^ myeloid cell number ratio, and TAMs with lower MHC-II expression despite treatment with APR-246 (Figure 3B). A more detailed analysis of the TME showed a similar T cell phenotype among the groups (Supplemental Figure 4, A and B). However, the TME from CSF1R-p53^KO^ mice revealed a reduced frequency of MHC-II (M1) and increased frequency of CD206 (M2) among myeloid cells with and without APR-246 treatment (Supplemental Figure 4C), and decreased frequencies of CD8^+^ T cells that were granzyme B^+^, Ki67^+^, and expressed immune checkpoints compared with those seen with p53-intact CSF1R-p53^WT^ T cells (Supplemental Figure 4, D and E). DCs also displayed a decrease in MHC-II and CD80 expression, suggesting decreased antigen presentation (Supplemental Figure 4F). Thus, lack of p53 in myeloid cells failed to induce a T cell–permissive TME with APR-246 treatment. Furthermore, CSF1R-p53^KO^ mice treated with APR-246 plus anti–PD-1 resulted in loss of tumor control and worse survival benefit compared with CSF1R-p53^WT^ mice treated with the same regimen (Figure 3C). Therefore, p53 activation in TME-associated myeloid cells is essential to augment the antitumor effects of anti–PD-1 therapy.

To study the effects of APR-246 mediated through p53 in T cells in the TME, we generated tissue-specific CD8- and p53-knockout mice (CD8-p53^KO^; CD8^cre^ × p53^fl^ crossbreeding). Non–Cre-expressing (CD8-p53^WT^) littermates were used as control. This conditional knockout causes loss of p53 in both CD4^+^ and CD8^+^ T cells since both are expressed during T cell development. T cells in the TME from vehicle-treated CD8-p53^WT^ and CD8-p53^KO^ mice had lower p53 levels, and APR-246 did not increase the levels of p53 in the TME of CD8-p53^KO^ mice. However, p53 levels in the TAMs from both CD8-p53^WT^ and CD8-p53^KO^ mice remained intact (Figure 3D). The TME from APR-246–treated CD8-p53^KO^ mice retained key T cell–facilitating features such as higher CD8^+^ T cell infiltration, a higher CD8^+^ T cell/CD11b^+^ myeloid cell number ratio, and TAMs with higher MHC-II expression (Figure 3E). A more detailed analysis of the TME showed only minor differences in APR-246– versus vehicle-treated mice in the phenotypes of T cells and myeloid cells from both CD8-p53^KO^ and CD8-p53^WT^ mice such as increased activated CD8^+^ T cells, a higher frequency of MHC-II (M1), and lower frequency of CD206 (M2) (Supplemental Figure 5, A–C and F). Compared with CD8-p53^WT^, APR-246–treated CD8-p53^KO^ mice revealed decreased frequencies of Tregs and proliferating Ki67^+^CD4^+^ T cells (Supplemental Figure 5D). On the other hand, CD8^+^ T cells from APR-246–treated CD8-p53^KO^ mice revealed increased cytotoxicity (granzyme B^+^) and proliferation (Ki67^+^), similar to those seen with p53-intact CD8-p53^WT^ T cells (Supplemental Figure 5E). We next investigated whether APR-246 in combination with ICB could mediate tumor control in CD8-p53^KO^ as well as CD8-p53^WT^ mice. CD8-p53^KO^ mice had intact tumor control and improved survival with APR-246 plus anti–PD-1, indicating that loss of p53 in T cells did not abrogate the therapeutic effect associated with a systemic increase in p53 expression (Figure 3F).

We next studied the effect of TAMs obtained from APR-246– versus vehicle-treated CSF1R-p53^KO^ and CSF1R-p53^WT^ mice on T cells. TAMs (CD45^+^CD11b^+^TCRβ^–^F4/80^+^) sorted from p53-intact CSF1R-p53^WT^ mice treated with APR-246 showed a higher expansion index of T cells compared with vehicle, while the expansion index of T cells cultured with TAMs from APR-246–treated CSF1R-p53^KO^ mice were not different from those treated with vehicle (Figure 3G). Thus, APR-246 reverses T cell suppression mediated by TAMs in a p53-dependent manner.

### Increased p53 expression in the TME leads to superior antitumor response to ICB.

**Figure 4:**
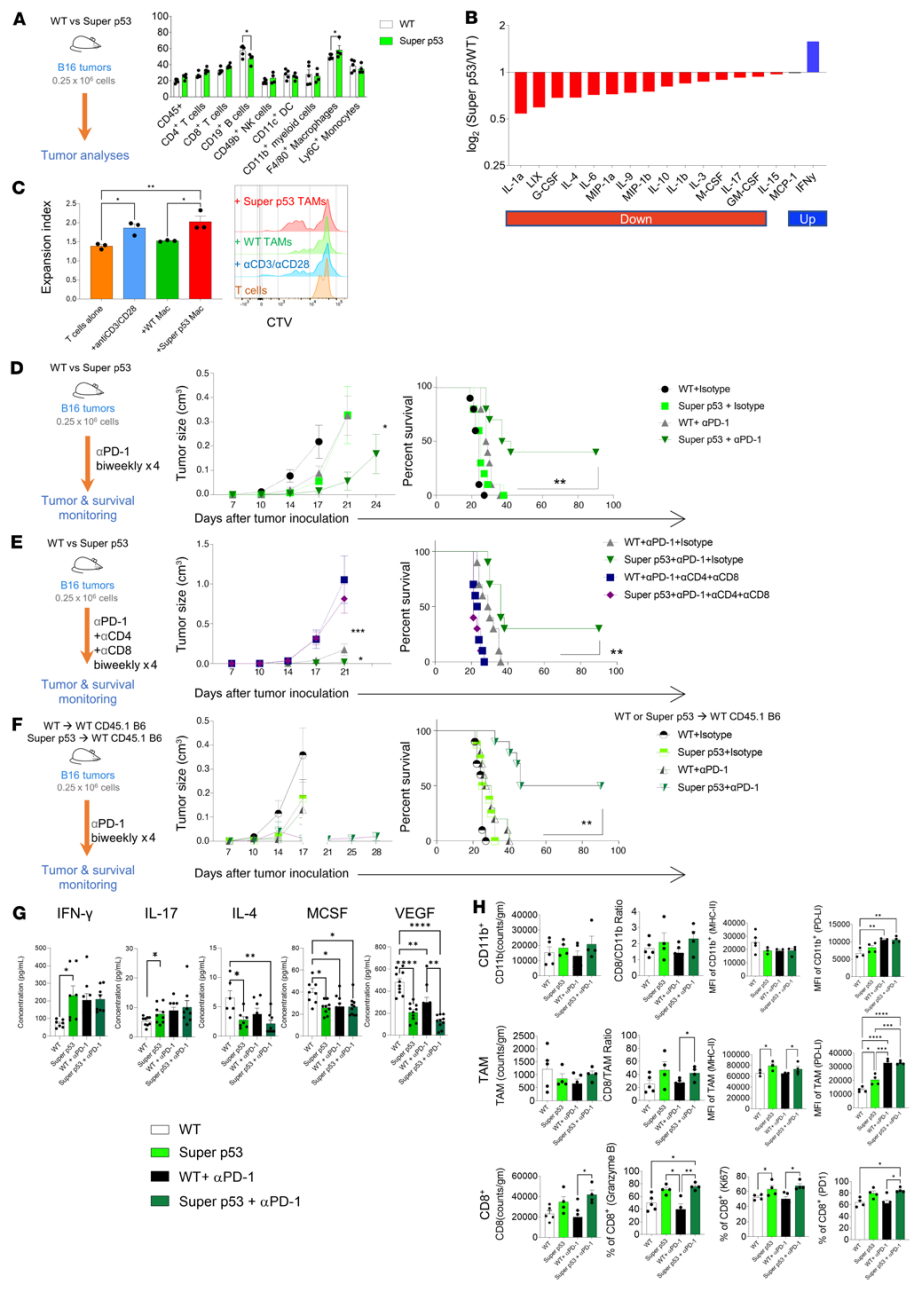
Increased p53 expression augments the antitumor effects of immune checkpoint blockade. (**A**–**C**) Schematic showing analysis of the TME of B16 tumors in super p53 versus WT mice. Tumors were harvested on day 13. (**A**) Flow cytometry was performed and frequency of p53^+^ events in gated immune subsets is depicted (*n* = 4–5/group, mean ± SEM shown). (**B**) Murine cytokine/chemokine array from tumor lysates from WT and super p53 mice. Bars represent the ratio of the average of intensity of 10 biological replicates (log_2_). (*n* = 9/group, performed in duplicate; mean ± SEM shown). (**C**) CD45^+^TCRβ^–^CD11b^+^F4/80^+^ TAMs were sorted from WT versus super p53 mice with B16 tumors. TAMs were cocultured with CTV-labeled CD8^+^ T cells that were magnetically sorted from non–tumor-bearing B6 mice as well as anti-CD3/anti-CD28–coated activating beads. CTV dilution was detected by flow cytometry and expansion index calculated by FlowJo. Plot is representative of 3 independent experiments. (**D**–**F**) Schematic of treatment groups, tumor growth curve, and Kaplan-Meier survival is depicted (plots depict 1 representative experiment, *n* = 9–10/group, performed in duplicate). (**D**) B16-bearing super p53 versus WT mice treated with and without anti–PD-1 antibody. (**E**) Super p53 versus WT mice inoculated with B16 melanoma and treated with anti–PD-1 antibody as well as anti-CD4/anti-CD8 depleting antibodies. (**F**) WT mice reconstituted with super p53 versus WT bone marrow inoculated with B16 melanoma and then treated with anti–PD-1 antibody. (**G** and **H**) Tumors from B16-bearing super p53 versus WT mice with and without anti–PD-1 antibody were harvested on day 13. (**G**) Murine cytokine array was performed on tumor lysates (*n* = 4–5/group, performed in duplicate; mean ± SEM shown) and (**H**) flow cytometry analyses enumerating CD11b^+^ myeloid cells, CD11b^+^F4/80^+^ TAMs and CD8^+^ T cells were performed (*n* = 4–5/group, mean ± SEM shown). *P < 0.05; **P < 0.01; ***P < 0.001; ****P < 0.0001 by 2-way ANOVA with multiple *t* tests corrected with Bonferroni’s method (**A** and **D**–**F** [tumor growth]), 1-way ANOVA with multiple *t* tests corrected with Bonferroni’s method (**C**, **G**, and **H**), or Kaplan-Meier with results ranked by Mantel-Cox log-rank test (**D**–**F** [survival patterns]).

While our data indicate that increased p53 expression using APR-246 augments the effects of ICB by reprograming the TME, it was unclear whether this mechanism of action was a direct consequence of increased p53 expression in the immune compartment of the TME independently from its activity on the tumor cells. To study the effects of increased p53 expression in immune cells of the TME on antitumor immunity, we utilized a transgenic mouse strain termed “super p53” that carries 2 copies of the *Trp53* transgene in addition to the 2 endogenous alleles (22). This results in an increased gene dosage of p53 and consequently an enhanced response to DNA damage, but retains normal regulation of p53. We implanted B16 melanoma tumor cells into both super p53 mice and WT littermate controls and performed flow cytometry analyses on the TME 2 weeks later (Figure 4A). In the super p53 TME versus WT TME, there was higher expression of p53 in the F4/80^+^ TAMs. In contrast to the TME from APR-246–treated animals, the increase was less prominent in other myeloid cell types. Chemokine and cytokine profiles of lysates from B16 tumors of super p53 and WT mice revealed a relative reduction predominantly in the M2-polarizing factors such as MIP-1, IL-1, IL-10, and IL-4 in the super p53 TME, and a relative increase in the M1-polarizing factor IFN-γ that is associated with T cell infiltration (Figure 4B). These effects were similar, but not identical, to the TME from mice treated with APR-246. WT CD8^+^ T cells cocultured with TAMs derived from super p53 hosts proliferated more than with those from WT, as indicated by a higher expansion index (Figure 4C). This result suggests that the TAMs in the TME from super p53 mice promote T cell expansion akin to those from APR-246–treated WT mice.

The data thus far indicate that increasing the genetic dosage of p53 in the TME can also induce a proinflammatory milieu that facilitates antitumor CD8^+^ T cells. We thus hypothesized that the super p53 TME, like the TME of APR-246–treated mice, would be responsive to ICB with anti–PD-1 antibody. We implanted super p53 and WT mice with B16 tumors and treated them with an anti–PD-1 antibody (RMP1-14) and monitored tumor progression and survival of mice (Figure 4D). There was a modest improvement in tumor control but no significant differences in survival between B16-bearing super p53 and WT mice treated with isotype control. However, tumor control in super p53 mice treated with PD-1 blockade was superior to WT controls, suggesting that enhanced p53 expression in the host adds to the activity of PD-1 blockade. Anti–PD-1–treated super p53 mice also exhibited longer overall survival. Differences in tumor control and survival between the super p53 and WT B16-bearing mice were lost upon T cell depletion using anti-CD4 (GK1.5) and anti-CD8 (2.43) antibodies, indicating that these effects are also T cell dependent (Figure 4E). To confirm the role of bone marrow–derived immune cells in the TME of the super p53 mice as opposed to stromal elements such as fibroblasts, we lethally irradiated WT recipients and reconstituted them with hematopoietic cells from super p53 or WT mice by syngeneic hematopoietic cell transplantation. After confirming full donor chimerism 3 months later, B16 tumors were implanted in these mice and the mice treated with anti–PD-1 (Figure 4F). WT mice reconstituted with super p53 bone marrow displayed longer survival with PD-1 blockade, whereas super p53 mice reconstituted with WT bone marrow had tumor control and survival with PD-1 blockade similar to those of WT mice reconstituted with WT bone marrow (Supplemental Figure 6). Taken together, these results confirm the importance of the role of extra gene dosage of p53 specifically in immune cells as opposed to stroma.

We next analyzed the TME of B16 melanoma tumors implanted in super p53 or WT mice treated with PD-1 blockade. The cytokine profile of the TME from super p53 mice showed higher levels of IFN-γ and decreased levels of M-CSF, VEGF (Figure 4G), IL-6, MCP-1, and MIP-2 (Supplemental Figure 7A). Analysis of the super p53 TME by flow cytometry revealed a significantly higher CD8^+^ T cell infiltration, in terms of both number and proportion (Figure 4H). The T cell compartment showed similar composition of CD4^+^ and CD8^+^ T cells, but CD8^+^ T cells displayed a lower proportion of CD62L^+^ and higher CD25^+^, indicating more activation (Supplemental Figure 7, B and C). The myeloid/macrophage cells had higher MHC-II expression, and a decreased proportion of CD206^+^ M2 cells, while the DCs were comparable (Figure 4H and Supplemental Figure 7, D and G). The CD4^+^ compartments were similar in the 2 groups, while the CD8^+^ T cells displayed greater cytotoxicity potential (granzyme B^+^), proliferation (Ki67^+^), and targets of immune checkpoint (PD-1^+^) (Supplemental Figure 7, E and F). We also saw a higher infiltration of melanoma-specific hGP100TCR^+^CD8^+^ T cells that were PD-1^+^ and CD44^+^ (Figure 4H and Supplemental Figure 7H). Addition of PD-1 blockade enhanced some T cell–facilitating features such as a decrease in VEGF and increased IL-17 and PD-L1 on TAMs. Thus, super p53 mice develop an immune-permissive TME that is similar but not identical to the TME of APR-246–treated mice, and that responds favorably to PD-1 blockade, resulting in better tumor control and longer survival.

### Increased p53 levels can affect canonical p53-associated functions.

**Figure 5:**
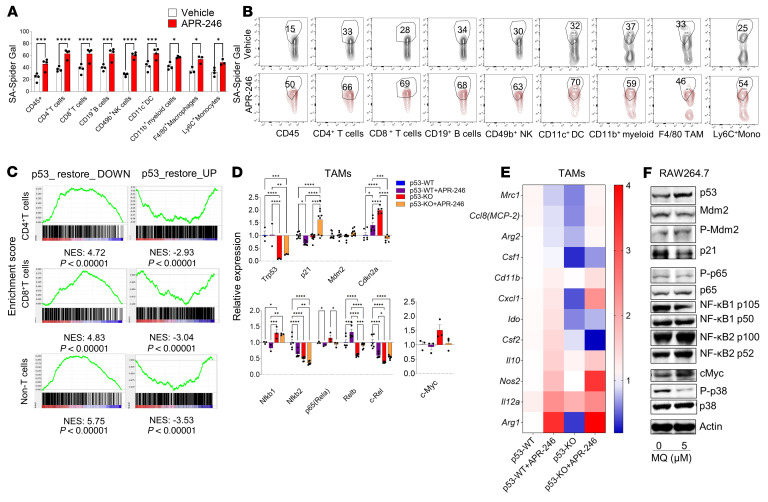
Increased p53 expression activates the SASP pathway. (**A**) Senescence-associated (SA) β-gal staining in different compartments of the TME in vehicle- and APR-246–treated mice (*n* = 5/group, mean ± SEM shown). (**B**) Representative plots of each immune subtype in the TME. (**C**) CD45^+^CD4^+^ T cells, CD45^+^CD8^+^ T cells, and non-T cells (CD45^+^CD4^–^CD8^–^) were sorted from the TME of B16 tumors in vehicle- or APR-246–treated mice, and RNA-seq was performed. GSEA plot evaluating changes in the p53 pathway depending on p53 expression (*n* = 3/group). (**D** and **E**) CD45^+^TCRβ^–^CD11b^+^F4/80^+^ TAMs were sorted from p53-WT (CSF1R-p53^WT^) versus p53-KO (CSF1R-p53^KO^) mice with or without APR-246 on day 13 of tumor growth and quantitative RT-PCR was performed (*n* = 3/group, done in triplicate; mean ± SEM shown). (**D**) Relative expression of genes of p53 signaling, NF-κB components, and c-myc. (**E**) Heatmap depicting expression of M1/M2 genes. (**F**) Western blotting was performed for key members of the p53, NF-κB, and MAPK pathways on RAW264.7 cells treated with MQ (blot representative of 2 experiments depicted). *P < 0.05; **P < 0.01; ***P < 0.001; ****P < 0.0001 by 2-way ANOVA with multiple *t* tests corrected with Bonferroni’s method (**A** and **D**).

The p53 protein is a tightly regulated molecule with several regulatory loops that function as a rheostat for its expression and functions (23). We therefore investigated whether increased p53 expression obtained by genetic or pharmacological means affects canonical p53-associated functions. A key function of p53 is mediated by induction of senescence, a state of cell cycle arrest without apoptosis. Senescent cells stain deeply with conventional senescence-associated spider β-galactosidase (SA-Spider-gal) substrate (10). Indeed, flow cytometry revealed higher frequencies of SA-spider-gal^+^ immune cells comprising lymphoid and myeloid subsets in APR-246–treated mice compared with vehicle-treated mice (Figure 5, A and B). The differences in SA-spider-gal^+^ immune cells in the super p53 versus WT TME were less pronounced, but significantly higher SA-Spider-gal^+^F4/80^+^ TAMs were found in the super p53 TME (Supplemental Figure 8, A and B).

For a comprehensive understanding of the molecular effects of APR-246 therapy on the immune infiltrate of the TME, we sorted CD4^+^ T cells, CD8^+^ T cells, and non-T cells (CD4^–^CD8^–^) from APR-246–treated WT mice and obtained global transcriptomic profiles using RNA sequencing (RNA-seq). We performed gene set enrichment analysis (GSEA) of 1093 curated genes that were upregulated and 613 curated genes that were downregulated by p53 restoration, as described previously (24). We found similarities with the differentially expressed upregulated and downregulated genes in the CD4^+^ T cells, CD8^+^ T cells, and non-T subsets of the TME (Figure 5C).

In parallel, we sorted CD4^+^ T cells, CD8^+^ T cells, and non-T cells (CD4^–^CD8^–^) from the TME of super p53 and WT mice, and similarly obtained a global transcriptomic profile by RNA-seq. As with the transcriptome of the APR-246–treated TME, we found similarities between the differentially expressed genes and those associated with p53 restoration (Supplemental Figure 8C). We also found that CD4^+^ and CD8^+^ T cells in super p53 mice showed upregulation of immune checkpoint genes like *Pdcd1* (PD-1) and *Lag3* (Lag-3) (Supplemental Figure 9, A and B). Non-T cells from the super p53 TME also showed downregulation of genes associated with M2 polarization (Supplemental Figure 9C). As the p53-induced SASP can influence the TME, we investigated whether increased p53 can affect the transcriptional program directly in the immune cells of the TME. We studied expression of the SASP gene set in the non-T cells of the TME, consisting of B cells and innate cells, including myeloid/macrophage cells (12). The TME of super p53 mice displayed differential expression of SASP genes, including those associated with M2 polarization and wound healing that can promote resistance to ICB therapy (Supplemental Figure 9D). We also found that the GSEA of the differentially expressed genes from non-T cells had significant similarities to those associated with the NF-κB pathway, a critical mediator of SASP (25) (Supplemental Figure 9E).

When comparing the curated set of genes regulated by p53 restoration, the differentially expressed genes in super p53 and APR-246–treated mice revealed remarkable similarities denoted by overlapping expression in those that are up- and downregulated (Supplemental Figure 10A). Further analysis of immune checkpoint and costimulatory markers in CD4^+^ and CD8^+^ T cells from the APR-246–treated TME also revealed upregulation of PD-1 and Lag-3 on T cells (Supplemental Figure 10, B and C). Examination of genes associated with M1 and M2 polarization in the non-T cell group also showed reduced expression of M2 genes (*Mrc1*, *Cox2*, and *Il10*) in the APR-246–treated tumors (Supplemental Figure 10D).

We recognized that the sorted non-T cells are heterogeneous and included B cells, NK cells, and myeloid cells, including TAMs. TAMs can suppress or support T cell proliferation and activity, as well as produce cytokines and chemokines that regulate inflammation in the TME that are important in antitumor immunity (9). Since we had already found decreased M2 cytokines and chemokines in the tumor lysates, and increased T cell proliferation with TAMs from APR-246–treated mice, we investigated the transcriptomic consequences of p53-regulated genes in TAMs after APR-246 therapy in sorted TAMs (CD45^+^CD11b^+^TCRβ^–^F4/80^+^) by quantitative real-time polymerase chain reaction (RT-PCR) (Figure 5D). TAMs from APR-246–treated mice displayed increased *Cdkn2a* and paradoxical decreases in *Mdm2* and *p21* (*Cdkn1a*). Increased *Cdkn2a* expression suggests transcriptomic induction of cellular senescence (26). Induction of *Cdkn2a* expression was lost in TAMs lacking p53 (CSF1R-p53^KO^). APR-246 treatment was associated with downregulation of NF-κB subunit p65 (*RelA*) and *cMyc*, both effectors that can control SASP. On the other hand, TAMs lacking p53 showed dysregulation of NF-κB subunits and c-Myc. Further, TAMs with intact p53 treated with APR-246 upregulated *Cxcl1*, *Nos2*, and *Il12* and downregulated *Ccl8* and *Mrc1*, suggesting transcriptomic suppression of M2 genes and upregulation of M1-associated genes (Figure 5E). The transcriptomic reprograming of TAMs induced by APR-246 was lost in mice lacking p53 (CSF1R-p53^KO^), indicating that the complete and efficient TAM polarization from M2 to M1, mediated by APR-246, was dependent on p53.

We similarly studied the genes directly regulated by p53 in TAMs from super p53 versus WT mice (Supplemental Figure 8D). Similar to that seen in TAMs from APR-246–treated mice, *p21* (*Cdkn1a*) and *Mdm2* genes were downregulated, while *Cdkn2a* was upregulated, suggesting induction of cellular senescence in TAMs from super p53 mice as well. In contrast to APR-246–treated TAMs, super p53–derived TAMs had increased p65 (*RelA*) expression but a similar decrease in c-Myc. These changes were associated with upregulation of *Cxcl1*, *Nos2*, and *Il12*, and downregulation of *Arg1*, *Ccl8* (MCP-2), *Mrc1* (CD206), and *Ido*, confirming transcriptional reprogramming of TAMs into ones with M1 features (Supplemental Figure 8E).

Despite a similar phenotype and transcriptional signatures, we found that the transcriptomic regulation of NF-κB subunits in TAMs from super p53 mice and APR-246–treated mice compared with controls were dissimilar. NF-κB is regulated posttranscriptionally and phosphorylation of its subunits is critical in NF-κB–directed transactivation (27). To further understand the regulation of p53-dependent SASP effects induced by APR-246, we treated monocyte/macrophage-like RAW 264.7 cells in vitro with methylene quinuclidinone (MQ), the active conversion product of APR-246 (28), and studied key components of the p53, NF-κB, and MAPK pathways by immunoblotting (Figure 5F). Treatment with MQ induced an increased p53 level, but similar decreases in total MDM2 and p21 levels. However, there was an increase in phosphorylated MDM2, and when taken together with the decrease in the total levels of MDM2 as well as the increase in p53 levels, is strongly indicative of increased stabilization and thus activity of p53. Immunoblots of the components of NF-κB subunits showed that despite modestly increased total p65, phosphorylated p65 that is a key controller of SASP was decreased. There was an increase in c-Myc, similar total p38 (MAPK14), and a decrease in phosphorylated p38, indicating suppression of the MAPK pathway. Thus, in monocytes/macrophages, APR-246 treatment increases p53 and activated p53–dependent regulation of MAPK and NF-κB pathways that in turn can control SASP. In vitro treatment of B16 cells with MQ also induced an increase in p53 and phosphorylated MDM2 (Supplemental Figure 11). This was associated with a similar p65 level and decreased phosphorylated p65. In contrast to RAW264.7, MQ treatment of B16 cells led to a decrease in c-Myc but similar levels of phosphorylated p38, suggesting distinct SASP regulation in the RAW264.7 macrophage and B16 melanoma cell lines. Collectively, these data indicate that genetically or pharmacologically increased expression of p53 in TAMs and can affect canonical p53-regulated pathways such as senescence and SASP.

### Responses to PD-1 blockade with APR-246 therapy are associated with a distinct immune signature in patients.

Our preclinical findings led to the development of a phase I/II clinical trial using APR-246 with ICB for patients with advanced solid tumors (ClinicalTrials.gov NCT04383938). In this ongoing trial, patients previously treated with ICB were treated with APR-246 and the PD-1–blocking antibody pembrolizumab. We obtained peripheral blood mononuclear cells (PBMCs) and sera from 2 of the patients with tumor regression and 2 patients in whom the tumors progressed on therapy (Supplemental Table 1). Biospecimens were collected at screening, prior to cycle 1, 2, and 5, and at the end of therapy in nonresponders. Analyses of PBMCs and sera at time points prior to therapy enabled us to study the sustained effects of reprogramed immune cells due to APR-246 and pembrolizumab, as opposed to transient changes.

**Figure 6:**
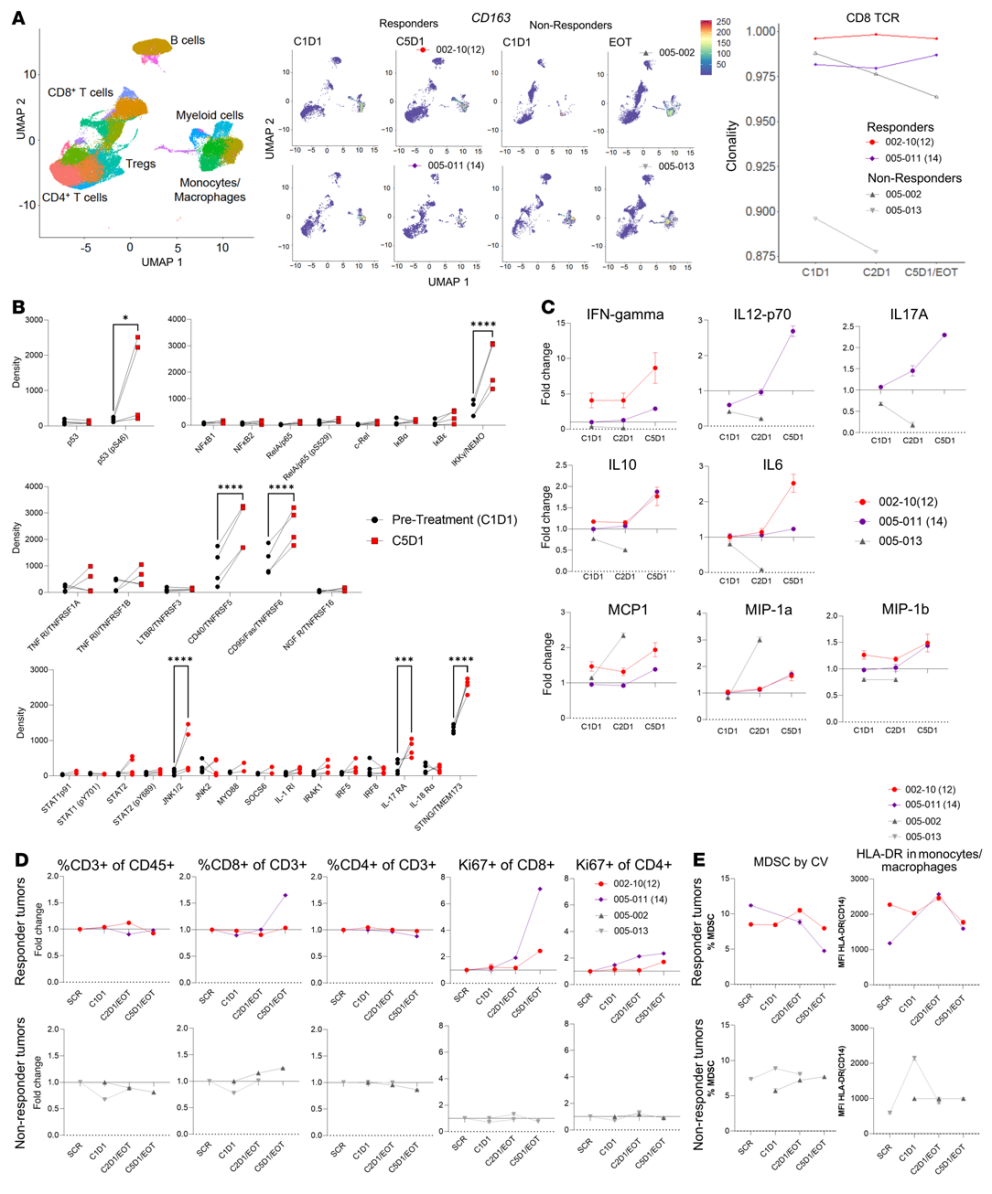
Treatment with the combination of anti–PD-1 and APR-246 reprograms the immune milieu in patients. PBMCs and serum were collected at the timing of screening (SCR), prior to cycle 1 day 1 (C1D1), cycle 2 day 1 (C2D1), cycle 5 day 1 (C5D1), or at the end of treatment (EOT) in nonresponders. Tumors of patients 002-10(12) and 005-11(14) displayed a reduction in size, while those from 005-02 and 005-13 continued to progress. (**A**) CITE-seq was performed on PBMCs, and subpopulations identified using UMAP. UMAP depicting CD163 expression in responders and nonresponders at C1D1 and C5D1 are depicted in the left panel. TCR clonality of CD8^+^ T cells is represented in the right panel. (**B**) Immuno-dot blotting was performed with PBMCs from 2 time points (prior to C1D1 and C5D1) from patients whose tumors responded [002-10(12) and 005-11(14)]. Density measured from immuno-dot blots of proteins are depicted (*n* = 2 × 2 technical replicates). *P < 0.05; ***P < 0.001; ****P < 0.0001 by 2-way ANOVA with multiple *t* tests corrected with Bonferroni’s method. (**C**) Cytokines in serum were quantified from 2 patients who responded and 1 who did not. Fold change in levels at different time points proportional to that at screening are depicted. (**D**) Flow cytometry was performed on PBMCs for T cell markers and fold changes in frequencies of populations at different time points proportional to that at screening are depicted. (**E**) MDSCs by coefficient of variation (CV) and HLA-DR expression on CD14^+^ myeloid cells were quantified.

We performed single-cell transcriptomics on the PBMCs using CITE-seq (cellular indexing of transcriptomes and epitopes by sequencing) and resolved the subpopulations using uniform manifold approximation and projection (UMAP) (Figure 6A and Supplemental Figure 12). The myeloid subpopulation, which included the macrophage/monocyte populations, showed a reduction in *CD163* (generally M2) myeloid populations prior to cycle 5 day 1 (C5D1) of treatment when compared with before therapy (C1D1) in patients with responses to APR-246 therapy. Concurrently, we found increased CD163^+^ myeloid cells in those who did not respond to APR-246 therapy. Additionally, genes associated with the cell cycle and those downregulated in induction of senescence were found to have decreasing *z* scores, while those upregulated in induction of senescence over time with therapy were found to increase (FRIDMAN SENESCENCE_PATHWAY) (Supplemental Figure 13). These results suggest induction of senescence by p53-activating APR-246 therapy, and a reduction in immunosuppressive M2 human monocyte/macrophage populations following systemic APR-246 therapy, similar to that seen in mouse TAMs. Concurrently, we also found that CD8^+^ T cells from the 2 responders maintained stably high TCR clonality (Figure 6A).

We next profiled key inflammation-associated proteins by immune-dot blotting in PBMCs from patients who responded, collected prior to initiation therapy and prior to C5D1 (Figure 6B). We found that there was increased p53 activation (phosphorylated S46) after therapy. We also found increased NEMO levels, suggesting regulation of NF-κB. This was associated with increased CD40, suggesting lymphocyte activation, and increased Fas levels, suggesting p53 activity. Importantly, there was increased inflammation that supports antitumor T cells, as indicated by levels of JNK1/2, IL-17RA, and STING. To further elucidate the effect of treatment with APR-246 over time on the immune milieu, we performed serum cytokine analysis and observed robust increases in T cell stimulating factors such as IFN-γ, IL-12 p70, and IL-17A in the responders, which was not seen in the patients whose tumors progressed (Figure 6C). However, in contrast to the data from the TME of APR-246–treated mice, IL-6 in serum only mildly increased in the responders on therapy. The myeloid/monocyte factors MCP-1 and MIP-1 did not increase significantly. Further T cell profiling of PBMCs by flow cytometry demonstrated strong proliferation (Ki67^+^) of CD4^+^ and CD8^+^ T cells in patients with tumor control (Figure 6D). In the patients with tumor control, flow-based profiling demonstrated a decrease in myeloid-derived suppressor cells (MDSCs) over time, while HLA-DR^+^ levels in myeloid cells remained relatively high (Figure 6E).

These data agree with our murine data and together illustrate changes in the myeloid compartment induced by APR-246 therapy as a mechanism to reprogram the TME and augment responses to ICB. While activated p53 was increased in responders after therapy, the patients with SASP induction in macrophages demonstrated T cell–facilitating properties and response to ICB. The ongoing clinical studies will potentially help determine biomarkers that are predictive of response to APR-246 plus ICB therapy.

## Discussion

Our data indicate that enhancing p53 either pharmacologically using APR-246 or by increasing its genetic dosage in the TME can augment the effects of ICB, leading to improved tumor control and increased survival in tumor-bearing mice. Increased p53 induces reprogramming of the TME into a T cell–facilitative environment by changing the cytokine expression, particularly in myeloid cells. We also show that increasing p53 expression in tumor-associated myeloid cells can induce canonical p53 effects such as senescence and p53-dependent regulation of MAPK and NF-κB pathways that control SASP.

APR-246 can structurally stabilize mutant p53 by reversibly binding to the core domain of the protein (16, 28), and it is postulated that in cells with WT p53, APR-246 binding results in an active and stable configuration of p53. Our data demonstrate that APR-246 can indeed increase WT p53 expression and thus influence the cells of the TME. Monotherapy with APR-246 did not affect growth of WT p53–expressing B16 tumors in mice but did alter the TME. The reprogrammed TME in turn augmented the response to ICB, leading to greater tumor control and longer survival.

There was an increase in p53 levels in a broader subset of immune cells in the TME of APR-246–treated than in super p53 mice, suggesting a tighter regulation of p53 in the transgenic mice. Accordingly, more senescent SA-Spider-gal^+^ cells were also seen in several immune subsets in the TME, compared with only in TAMs seen in the super p53 TME. This could be due to several reasons, including differences in the pharmacological activation of p53 and genetically enhanced p53. In addition, APR-246 treatment can affect tumor cells directly. Indeed, we found differences in the inflammatory milieu between APR-246–treated mice and super p53 mice, when compared with respective controls. However, treatment with APR-246 increased p53 in the myeloid cells in the TME and resulted in a p53-associated transcriptional programing similar to that seen in super p53 mice. Importantly, the therapeutic effects of APR-246 in combination with anti–PD-1 antibody was lost in mice lacking p53 in the myeloid cells but was retained in mice with p53-null T cells. These data indicate that SASP induced by enhanced p53 in the myeloid cells is key in generating a microenvironment that is permissive to T cell activation.

The immunoregulatory features of p53 signaling in tumors can affect development and progression of cancers. In colorectal carcinoma, signaling from mutated p53 promotes NF-κB activation and increases chronic inflammatory cytokine production (29). In breast cancer, loss of p53 increases systemic inflammation (30). Restored p53 in tumor cells can also provoke immune surveillance (31). Indeed, small molecule inhibitors of MDM2 increase p53 effects in tumor cells, and attenuate immune-inhibitory SASP to potentiate response to ICB (32). In these studies, the activity of p53 was primarily in the tumor cells, which then influenced the TME. Our data reveal that modulating p53 directly in the myeloid cells improves T cell activity in the context of ICB therapy. Increasing p53 in myeloid cells was adequate and critical to reprogram the TME to a pro-immune microenvironment. Increased p53 activity was associated with decreased IL-1β and inhibition of IL-1β is known to potentiate tumor control with ICB (33). Increased p53 expression in TAMs was also associated with upregulation of *Il12* and *Cxcl1* (typically associated with T cell potentiation) and downregulation of *Ccl8* and *Mrc1* (associated with chronic inflammation and T cell suppression). These genes are also known components of a p53-dependent senescence program (10). SASP factors reinforce the senescence program and influence the tissue microenvironment via cooperation of p53-controlled pathways, including NF-κB–dependent signaling, specifically the p65 subunit (25). Activation of p53 in cells that are exposed to genotoxic stress also induces senescence and SASP factors (34). Our data from cytokine arrays and RNA-seq suggested that most of the SASP markers are repressed following p53 augmentation, and only a small number of SASP genes that are induced by genotoxic stress are upregulated by increased p53. Consequently, inflammatory factors such as IL-1α and IL-1β were decreased in the TME of super p53 and APR-246–treated WT mice. This is not surprising, as p53 itself can transcriptionally repress some of the SASP genes that are associated with genotoxic agents, and therefore some of the detrimental effects of conventional SASP such as chronic inflammation are subdued (32, 35). Interestingly, even though IL-6 was significantly decreased following APR-246 therapy in mice, it appeared to increase in patients who responded to the therapy. This observation suggests that while certain p53-related functions in human immune cells are not conserved in mice (36), the overall effects on chronic inflammation are conserved. Our results are also concordant with previous reports that show p53, MAPK, and the NF-κB axes in macrophages are involved in the development of an M2 polarization process (37, 38). Our data are also concordant with recent data that show that the SASP programs induced with NF-κB, such as those associated with chemotherapies and genotoxic stress, induce an activation of protumorigenic inflammation, while a partial SASP program enriched with p53 targets promotes an inflammatory program that is more conducive to antitumor immunity (14). Thus, increasing p53 induces senescence but mostly downregulates inflammatory factors known to polarize M2 macrophages and promote immunotherapy resistance, while enhancing M1 polarization that facilitates T cell activity. An inflammatory TME is also associated with increased expression of PD-1 in T cells and its ligand PD-L1 in myeloid cells, which provide targets for ICB.

We used several aggressive tumor models that are known to respond poorly to PD-1 monotherapy. Hence it is not surprising that the combination of PD-1 and APR-246 led to a significant but modest improvement in overall survival. The combination was substantially better because the APR-246 treatment induced higher expression not just of the targets of anti–PD-1 but also CTLA-4 and other immune checkpoints. A more realistic estimation of clinical impact is unarguably obtained through a clinical trial, and indeed the combination of APR-246 with PD-1 blockade is currently being studied in a phase I/II clinical trial (ClinicalTrials.gov NCT04383938). Here, we depict preliminary findings from 2 patients with tumors that had stopped responding to anti–PD-1 and whose tumors decreased after combined treatment with APR-246 and pembrolizumab. We studied the PBMCs and sera from patients in this trial and found that despite known differences in certain p53-related function between mouse and human cells, the features of p53-induced senescence and regulation of MAPK and the NF-κB axes in myeloid populations induced by APR-246 therapy is relevant in humans. This was associated with suppression of CD163^+^ M2 monocytes/macrophages and CD8^+^ T cell expansion in patients who responded to the combination of APR-246 and ICB. The changes in the myeloid compartment also demonstrated a decrease in MDSCs and increased HLA-DR. While these differences were subtle in the peripheral blood, they were associated with stronger changes in the tumor milieu. Although we were able to obtain only very limited samples because the trial is still in progress, our data suggest that treatment with APR-246 plus ICB can potentiate antitumor T cells in patients with meaningful responses.

Thus, increased p53 expression in myeloid cells of the TME augments the antitumor effects of ICB. Reprogramming the TME by increasing p53 with APR-246 represents a potentially novel approach to combination immunotherapy with ICB. Beyond the direct effect on tumors, our study highlights the importance of the effect of p53 on the TME and provides rationale for combining such therapies with ICB. These data serve as the basis for more studies on p53-targeting molecules such as APR-246 and genotoxic therapies that further activate p53 to be used in combination with ICB.

## Methods

### Patient characteristics and analyses of human samples.

Data from biospecimens obtained from 4 patients are depicted here, who were enrolled sequentially into an ongoing phase I/II clinical trial (ClinicalTrials.gov NCT04383938). PBMCs and serum samples were collected at screening, prior to cycle 1 day 1, cycle 2 day 1, cycle 5 day 1, and at the end of treatment. The detailed methodologies are described in the Supplemental Methods.

### Mice.

All mice or progenitors of colonies were purchased from The Jackson Laboratory. C57BL/6J (B6) and Ly5.1 (CD45.1) mice were obtained at 6–8 weeks old and have been described previously (9). B6;CBA-Tg(Trp53)1Srn/J (super p53 mice) were backcrossed more than 10 generations with C57BL/6J mice, and non–transgene-expressing littermates used as WT controls. B6.129P2-Trp53tm1Brn/J (p53^fl^) (39) mice were crossbred with C57BL/6-Tg (CD8-cre)1Itan/J (CD8^cre^) mice (40) to generate CD8-p53^KO^ mice, and crossbred with C57BL/6-Tg(Csf1r-cre)1Mnz/J (CSF1R^cre^) mice (41) to generate CSF1R-p53^KO^ mice. Non–cre-bearing littermates were used as controls: CD8-p53^WT^ and CSF1R-p53^WT^.

### Tumor models and bone marrow chimeras.

The murine cancer cell line for melanoma (B16F10, referred to as B16) was obtained from ATCC, the colon cancer cell line MC38 was obtained from Kerafast and have been previously described (42–44). The TC1 HPV cell line, which was originally derived from primary epithelial cells of C57BL/6J mice cotransformed with HPV-16 E6 and E7 and c-Ha-ras oncogenes, was a gift from Dmitriy Zamarin (Memorial Sloan Kettering Cancer Center) (45). The cell lines have been tested as mycoplasma negative. Cells were maintained in RPMI medium supplemented with 10% fetal calf serum (FCS) and penicillin with streptomycin (complete RPMI media). Flank tumors were established according to the cell dose and schedule indicated in each figure to monitor for therapeutic efficacy and study the TME. Unilateral flank tumors were established by implantation of 2.5 × 10^5^ cells into the right flank for experiments to monitor for therapeutic efficacy, or 2.5 × 10^6^ cells into both flanks for TME evaluation. Tumor growth and survival were measured by calipers twice weekly, and mice with tumors measuring greater than 2.0 cm in any diameter, with ulceration, or in morbid condition were promptly euthanized. To generate bone marrow chimeras, recipient mice that were lethally irradiated with 1100 cGy were reconstituted with bone marrow from donors. The types of donors are depicted with each experiment. After 90 days, chimerism was confirmed and tumor challenge experiments performed.

### In vivo treatments.

APR-246 was provided by Aprea Therapeutics under a material transfer agreement. APR-246 was reconstituted in PBS just prior to injection and used at 100 mg/kg per mouse administered intraperitoneally (i.p.) daily as depicted in respective experiments; PBS was used as vehicle control. Therapeutic in vivo monoclonal antibodies (mAbs) anti–PD-1 (RMP1-14) and anti–CTLA-4 (9D9), corresponding IgG isotype controls (2A3 and MPC-11), and depleting mAbs anti-CD4 (GK1.5) and anti-CD8 (2.43) and their IgG isotype controls (LTF-2) were purchased from Bio X Cell. RMP1-14 (250 μg) and 2A3 (250 μg) were administered i.p. twice weekly beginning on day 7 for up to 4 doses. Depleting mAbs GK1.5 (560 μg) and 2.43 (400 μg) were administered i.p. twice weekly beginning on day 7 for 4 doses.

### Mouse tumor processing for flow cytometry.

Tumors were first weighed and then minced with scissors in RPMI and filtered through a 70-μm nylon filter (BD Biosciences) in RPMI to generate single-cell suspensions. The suspensions were purified on a Ficoll gradient to eliminate dead cells and treated with red blood cell lysis buffer (ACK Lysing Buffer, Lonza) and further washed and resuspended in FACS buffer (PBS/0.5% albumin) before incubation with antibodies. The antibodies used for staining are listed in Supplemental Table 2. In specified experiments, cell numbers were calculated per gram of tumor, and the ratio of CD8 to CD11b calculated from the absolute number of cells/gram of tumor. In experiments to detect SA-Spider-gal by flow cytometry, Spider β-Gal kits (Dojindo Laboratories) were used per the manufacturer’s protocol, prior to staining with antibodies. In experiments to study TCRs, tetramers of the following epitopes were used: hGP100_25–33_ (KVPRNQDWL)–Alexa Fluor 488 and OT-1 SIINFEKL-PE (NIH). Samples were acquired on a Cytek Aurora flow cytometer. Data analyses were performed using FlowJo v10 (FlowJo).

### In vitro T cell suppression/proliferation assay.

For the suppression assays with TAMs, single-cell suspensions of the tumors were obtained as described above and tumor-isolated macrophages (CD45^+^CD11b^+^F4/80^hi^Ly6G^–^) were sorted using a BD FACSAria at over 90% purity. CD8^+^ T cells isolated from the spleen of naive mice were purified using anti-CD8 (Ly-2) microbeads (Miltenyi Biotec) according to the manufacturer’s protocol. CD8^+^ T cells were then stained with CellTrace Violet (CTV) proliferation dye (Thermo Fisher Scientific) and plated in complete RPMI media supplemented with 0.05 M β-mercaptoethanol onto round-bottom 96-well plates (1 × 10^5^ cells per well) and were stimulated with anti-CD3/anti-CD28 microbeads (Dynabeads Mouse T-Expander CD3/CD28, Thermo Fisher Scientific). CD8^+^ T cells were plated with FACS-isolated TAMs and analyzed for CFSE dilution by flow cytometry. Expansion index was determined using FlowJo v10.

### Quantitative RT-PCR AND RNA-seq.

For RNA-seq, mice bearing B16 tumors were implanted in super p53 or WT mice. WT mice were treated with vehicle or APR-246 as described starting on day 7 for 6 days and single-cell suspensions of the tumors were obtained and stained with antibodies for CD45, CD8, CD4, and live/dead as described above. CD45^+^CD4^+^, CD45^+^CD8^+^, and CD45^+^CD4^–^CD8^–^ cells were sorted and RNA was extracted with the PAXgene RNA kit and purified in accordance with the manufacturer’s protocols. RNA was assessed with the Agilent BioAnalyzer for quantity and quality. For library preparation, we used the Globin-Zero kit (EpiCentre) and the Illumina TruSeq mRNA Stranded Library kit, with 11 to 12 PCR cycles for 5 to 8 nmol/L input, and sequencing was performed on a HiSeq 2500 system (Illumina) with 150-bp paired-end reads. Library was prepared and RNA-seq was done at Expression Analysis (Q2 Solutions). Approximately 10 million 100-bp single-end reads were retrieved per replicate condition. Resulting RNA-seq data were analyzed by removing adaptor sequences using Trimmomatic (46), aligning sequencing data to GRCm38.91(mm10) with STAR, and genome-wide transcript counting using featureCounts (47) to generate an RPKM matrix of transcript counts. Genes were identified as differentially expressed using R package DESeq2 with a cutoff of absolute log_2_(fold change) of 1 or greater and adjusted *P* value of less than 0.05 between experimental conditions (48). For heatmap visualization, samples were *z*-score normalized and plotted using the pheatmap package in R. Functional enrichments of these differentially expressed genes were performed with enrichment analysis tool enrichR (49) and the retrieved combined score (log[*P* value] × *z* score) are displayed. The RNA-seq data have been deposited in NCBI’s Gene Expression Omnibus (GEO) (accession: https://www.ncbi.nlm.nih.gov/geo/query/acc.cgi?acc=GSE166637).

For RT-PCR, mice bearing B16 tumors were implanted in super p53 or WT mice. In separate experiments, B16 tumors were implanted in CSF1R-p53^WT^ and CSF1R-p53^KO^ mice, treated with vehicle or APR-246 as described above starting on day 7 for 6 days, and single-cell suspensions of the tumors were obtained and stained for live/dead, CD45, CD8, CD4, TCRβ, CD11b, and F4/80 as described above. CD45^+^TCRβ^+^CD8^+^ T cells, CD45^+^TCRβ^+^CD4^+^ T cells, and CD45^+^TCRβ^–^CD11b^+^F4/80^+^ TAMs were sorted into tubes with TRIzol LS (Thermo Fisher Scientific) and frozen. Total RNA extraction was processed following the manufacturer’s instructions and 100–500 ng of total RNA was reverse transcribed into first-strand cDNA with a High-Capacity cDNA Archive Kit (Thermo Fisher Scientific). RT-PCR was performed using PreAmp master mix (Fluidigm) and the mixtures of all the Taqman assays (Thermo Fisher Scientific) using an MX IFC controller (Fluidigm), and expression data were collected by BioMark HD System. The mRNA levels were normalized to *Gapdh* and are reported as relative mRNA expression using the equation ΔΔ*C_t_* = 2^–(Δ*Ct*sample^^–^^Δ*Ct*control)^.

### Mouse cytokine multiplex.

Tumors were excised, snap-frozen, and homogenized using a tissue grinder. Total protein concentration was normalized to 50 ng/mL. Cytokines were quantified using the MILLIPLEX MAP Mouse Cytokine/Chemokine Magnetic Bead 32 Plex Panel according to the manufacturer’s instructions (Millipore).

### Immunoblotting.

B16 cells or RAW cells were plated in 10- to 15-cm tissue culture dishes in complete RPMI media. Twenty-four hours later, they were treated with MQ (concentration as indicated) in RPMI media devoid of nonessential amino acids for 4 hours. The cells were harvested 24 hours after treatment and total protein lysates were prepared using RIPA buffer (Cell Signaling Technology, catalog 9806S) supplemented with protease and phosphatase inhibitors (Thermo Fisher Scientific). Protein lysates were separated on precast 4%–12% polyacrylamide NuPAGE Novex Bis-Tris gels (Invitrogen) and then electrotransferred onto PVDF membranes (EMD Millipore). The membranes were blocked and incubated with the respective primary and secondary antibodies. The antibodies used are listed in the Supplemental Methods. Chemiluminescence was detected with the Western Lighting Plus ECL (PerkinElmer) or SuperSignal West Pico reagent (Thermo Fisher Scientific/Pierce) using the Invitrogen iBright FL1000 imaging system.

### Statistics.

Data were analyzed for statistical significance with an unpaired, 2-tailed Student’s *t* test when comparing the means of 2 independent groups or ANOVA (for comparisons of ≥3 groups), as appropriate. All data represent the mean ± SEM. In experiments with multiple *t* test correction, *P* values were adjusted using Bonferroni’s method. Evaluation of survival patterns in tumor-bearing mice was performed by the Kaplan-Meier method, and results were ranked according to the Mantel-Cox log-rank test. Survival was defined as otherwise healthy-appearing mice with nonulcerated size-limited tumors. A *P* value of less than 0.05 was considered statistically significant.

### Study approvals.

Mouse experiments were performed in accordance with institutional guidelines under a protocol approved by the Memorial Sloan Kettering Cancer Center Institutional Animal Care and Use Committee. All mice were maintained in a pathogen-free facility according to the NIH *Guide for the Care and Use of Laboratory Animals* (National Academies Press, 2011). Patients were enrolled in a multicenter, open-label, dose-finding and expansion study of eprenetapopt (APR-246) in combination with pembrolizumab in advanced or metastatic solid tumors (ClinicalTrials.gov NCT04383938), conducted at 9 academic research hospitals in the United States: Mayo Clinic, Jacksonville, Florida; Mayo Clinic, Phoenix, Arizona; Mayo Clinic, Rochester, Minnesota; Massachusetts General Hospital, Boston, Massachusetts; Washington University, St. Louis, Missouri; Vanderbilt University, Nashville, Tennessee; and the MD Anderson Cancer Center, Houston, Texas. The trial was conducted according to principles of the Declaration of Helsinki, Good Clinical Practice, and applicable regulatory requirements. The protocol, consent procedures, and any amendments were approved by the relevant institutional review boards or ethics committees. All patients provided written informed consent before study participation. Patient biospecimens were collected on a tissue-collection protocol approved by the relevant institutional review boards. Deidentified cryopreserved samples were shipped from a central biostorage facility for experimentation.

## Author contributions

AG, JM, SWL, TM, and JDW developed the concepts. AG, RM, SB, AC, LFC, RZ, and ODH designed experiments and analyzed data. RM, DV, LD, and KF performed and analyzed animal model experiments, flow cytometry experiments, and functional assays. JF and CL performed molecular biology experiments. YJH, LM, and AC performed bioinformatics analysis. RG, FS, NS, AH, MAA, YB, and HZ provided technical assistance and KSP provided biostatistical assistance. AG, RM, DV, LD, MR, SWL, TM, and JDW wrote the manuscript. We determined the first coauthorship order by evaluating the key contribution of each author, and the first coauthorship was determined by the fact that the 2 first authors have been key contributors to the findings described in this manuscript.

## Supplementary Material

Supplemental data

## Version 1 09/15/2022

Electronic publication
