# Supplementary material for: Increased p53 expression induced by APR-246 reprograms tumor-associated macrophages to augment immune checkpoint blockade
Source: J Clin Invest. 2022 Sep 15;132(18):e148141. doi: 10.1172/JCI148141 (PMC9479603; doi:10.1172/JCI148141)
Supplement: Supplemental data [file jci-132-148141-s009.pdf]

Supplementary Methods

Single Cell RNAseq: For Cellular Indexing of Transcriptomes and Epitopes by sequencing (CITE-Seq), PBMC samples were thawed, stained with DAPI and live cells sorted using FACSAria (BD Biosciences). The cells were then barcoded by staining with TotalSeq™-C Universal Cocktail v1.0 (Biolegend) and following quality checks, single cell RNA expression and protein sequencing assay performed as described (1) using following 10x Genomics Single Cell protocol with VDJ enrichment (10xGenomics). Single-cell sequencing data was aligned to the Genome Reference Consortium Human Build 38 (GRCh38) using Cell Ranger (v6.1.1; 10X Genomics) in order to obtain T cell clonotypes, feature barcoding, CITE-seq antibody detection and gene expression profiles associated with individual single-cells. Each datatype was matched to create a UMI matrix and cells were filtered out based on three metrics: (1) cells with less than 200 detectable genes; (2) cells with more than 3500 detectable genes; (3) cells that had less than 8% percentage of counts related to mitochondrial genes. Data normalization, Principal Component Analysis, Uniform Manifold Approximation and Projections (UMAP), downstream statistics and figure generation was performed on the dataset using the R package Seurat v.3.1.1 (https://github.com/satijalab/seurat). Differential expression comparisons were generated using the DESeq2 package with selected genes (FDR < 0.05). For supervised annotation of single cells, after filtering, we created subclusters of cells using the Louvain algorithm (2). Clusters of immune subsets were manually annotated based on known markers. For TCR clonality we used the Shannon-Weiner diversity index.

Serum cytokine: For cytokine levels, serum samples were thawed, and cytokine concentrations quantified using Luminex-based bead multiplex immunoassays according to the manufacturers' instructions (Millipore).

Flow cytometry: For flow cytometry, PBMC samples were thawed and stained with a fixable Aqua viability dye (Invitrogen) and a cocktail of antibodies to the following surface markers was used: CD8-Qdot605 (Invitrogen, 3B5), CD4-Qdot 655 (Invitrogen, S3.5), PD-1-PE (BD, MIH4), LAG-3-FITC (Enzo, 17B4), ICOS-PE-Cy7 (eBioscience, ISA-3), TIM-3-APC (R&D Systems, 344823). Cells were next fixed and permeabilized with the FoxP3/Ki-67 Fixation/Permeabilization Concentrate and Diluent (eBioscience), and subsequently stained intracellularly with CD3-BV570 (Biolegend, UCHT1), Ki-67-AlexaFluor700 (BD, B56), FoxP3-eFluor450 (eBioscience, PCH101), and CTLA-4-PerCP-eFluor710 (eBioscience, 14D3). For characterization of myeloid cells including myeloid-derived suppressor cells (MDSCs) a cocktail of antibodies to the following surface markers was used: CD14-PerCP-Cy5.5 (BD Biosciences, M5E2), HLA-DR-ECD (Beckman Coulter, Immu-357), Lineage cocktail – CD3/CD16/CD19/CD20/CD56-FITC (BD Biosciences, SK7/3G8/SJ2 5C1/L27/NC AM16.2). Stained cells were acquired on a LSRFortessa (BD Biosciences) and analyzed using FlowJo software (FlowJo, LLC). CD14+HLA-DRlo monocytic MDSC frequencies were derived objectively by gating on live, lineage-negative CD14+ monocytes and exporting this population to a computational algorithm based on the monocyte HLA-DR mean fluorescence intensity coefficient of variation and interpolation from MDSC frequencies obtained from the HLA-DR CV spread of CD14+ monocytes of healthy human donors (3).

Immune dot blot: For immune dot blot, we performed Human NFκB Pathway Array with frozen PBMC samples as described in the manufacturer's protocol (RnD systems Catalog Number ARY029).

List of antibodies for western blots: β-actin (clone C4, Santa Cruz Biotechnology, cat #sc-47778), p53 (clone 1C12, Cell Signaling Technology, cat #2524s), p21(Cell Signaling Technology, cat #64016s), Mdm2 (clone E3g51, Cell Signaling Technology, cat #51541s), p-Mdm2 (S166) (Invitrogen, cat #44-1400g), p65 (clone D14E2, Cell Signaling Technology, cat# 8242s), p-p65 (S536) (clone 93H1, Cell Signaling Technology, cat# 3033s), p38 (clone D13E1, Cell Signaling Technology, cat# 8690s), p-p38 (T180/Y182) (clone D3F9, Cell Signaling Technology, cat# 4511s), NFKB1 (clone D4P4D, Cell Signaling Technology, cat# 13586s), NFKB2 (clone D9S3M, Cell Signaling Technology, cat# 52583s), cMyc (clone D84C12, Cell Signaling Technology, cat# 5605s).The corresponding secondary IgG antibodies conjugated to horseradish peroxidase were obtained from Signa Aldrich.

Supplemental Reference

1. Stoeckius M, Hafemeister C, Stephenson W, Houck-Loomis B, Chattopadhyay PK, Swerdlow H, et al. Simultaneous epitope and transcriptome measurement in single cells. *Nat Methods.* 2017;14(9):865-8.
2. Lancichinetti A, and Fortunato S. Community detection algorithms: a comparative analysis. *Phys Rev E Stat Nonlin Soft Matter Phys.* 2009;80(5 Pt 2):056117.
3. Kitano S, Postow MA, Ziegler CG, Kuk D, Panageas KS, Cortez C, et al. Computational algorithm-driven evaluation of monocytic myeloid-derived suppressor cell frequency for prediction of clinical outcomes. *Cancer Immunol Res.* 2014;2(8):812-21.

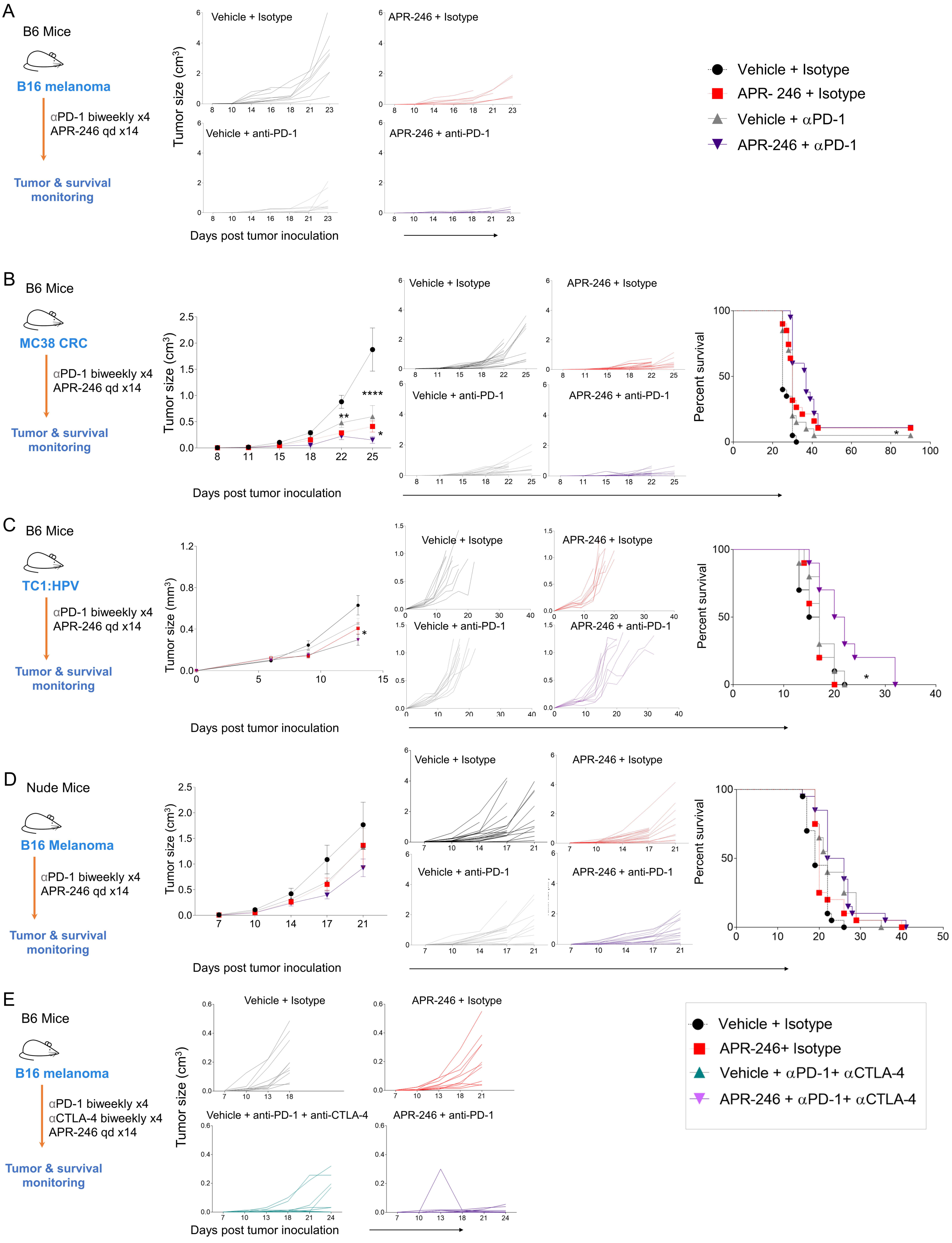

Supplemental Figure S1: (A) Schematic of treatment groups and individual tumor growth curves of the results depicted in Fig 1D. Schematic of treatment groups, tumor growth curve and Kaplan Meier survival of (B) APR-246 vs. control with and without anti-PD in B6 mice with MC38 tumors (C) APR-246 vs. control with and without anti-PD1in B6 mice with TC1:HPV tumors and (D) APR-246 vs. control with and without anti-PD1 in nude mice with B16 tumors. (E) Schematic of treatment groups and individual tumor growth curves of the results depicted in Fig 1E.

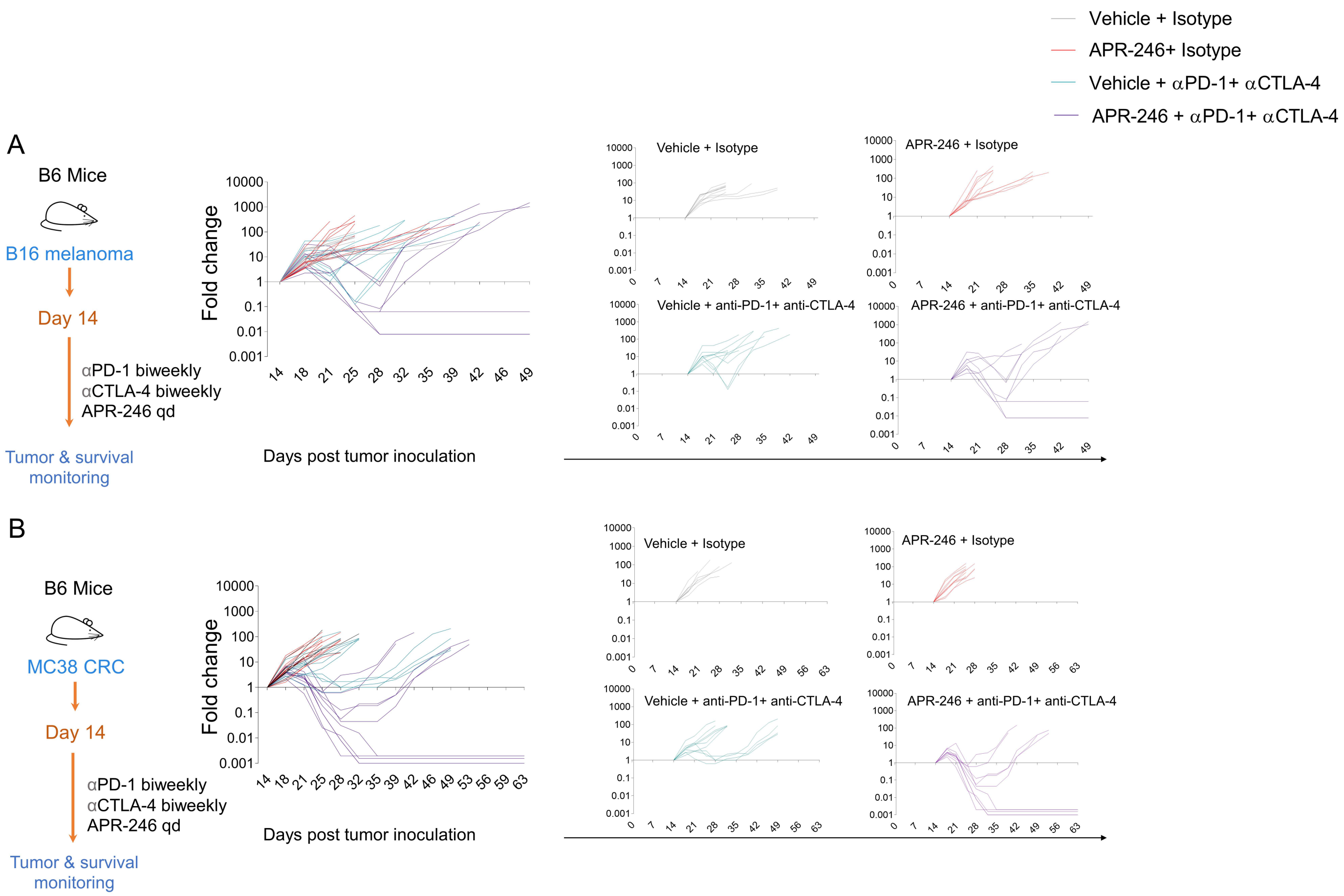

Supplemental Figure S2: Experiments depicted in Figure 1F, G (A) Schematic of treatment, fold changes (vs pretreatment) in tumor growth of APR-246 vs. control, with and without anti-PD1 and anti-CTLA-4, and individual tumor growth patterns of groups of B6 mice with B16 tumors represented groupwise **(B)** Schematic of treatment, fold changes (vs pretreatment) in tumor growth of APR-246 vs. control, with and without anti-PD1 and anti-CTLA-4, and individual tumor growth patterns of groups of B6 mice with MC38 colorectal carcinoma (CRC) tumors

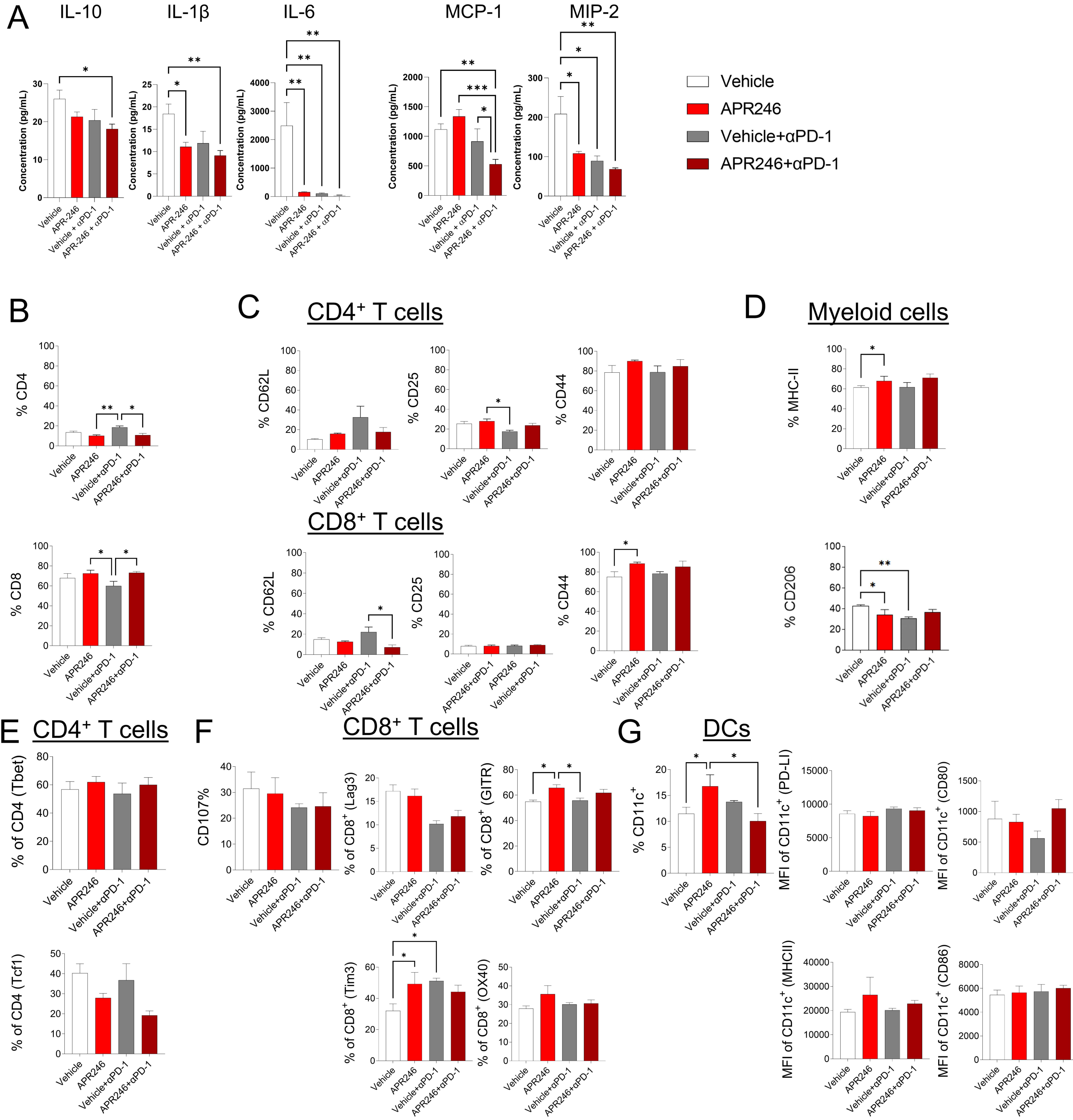

Supplemental Figure S3 (A) Murine cytokine array as performed on tumor lysates (N=4-5/group, performed in duplicates; mean with SEM shown) **(B-G)** Multicolor flow cytometry analyses of live $CD45^+$ gated cells of the TME. (N=5/group mean with SEM shown) (*P < 0.05; **P < 0.01; ***P < 0.001; ****P < 0.0001)

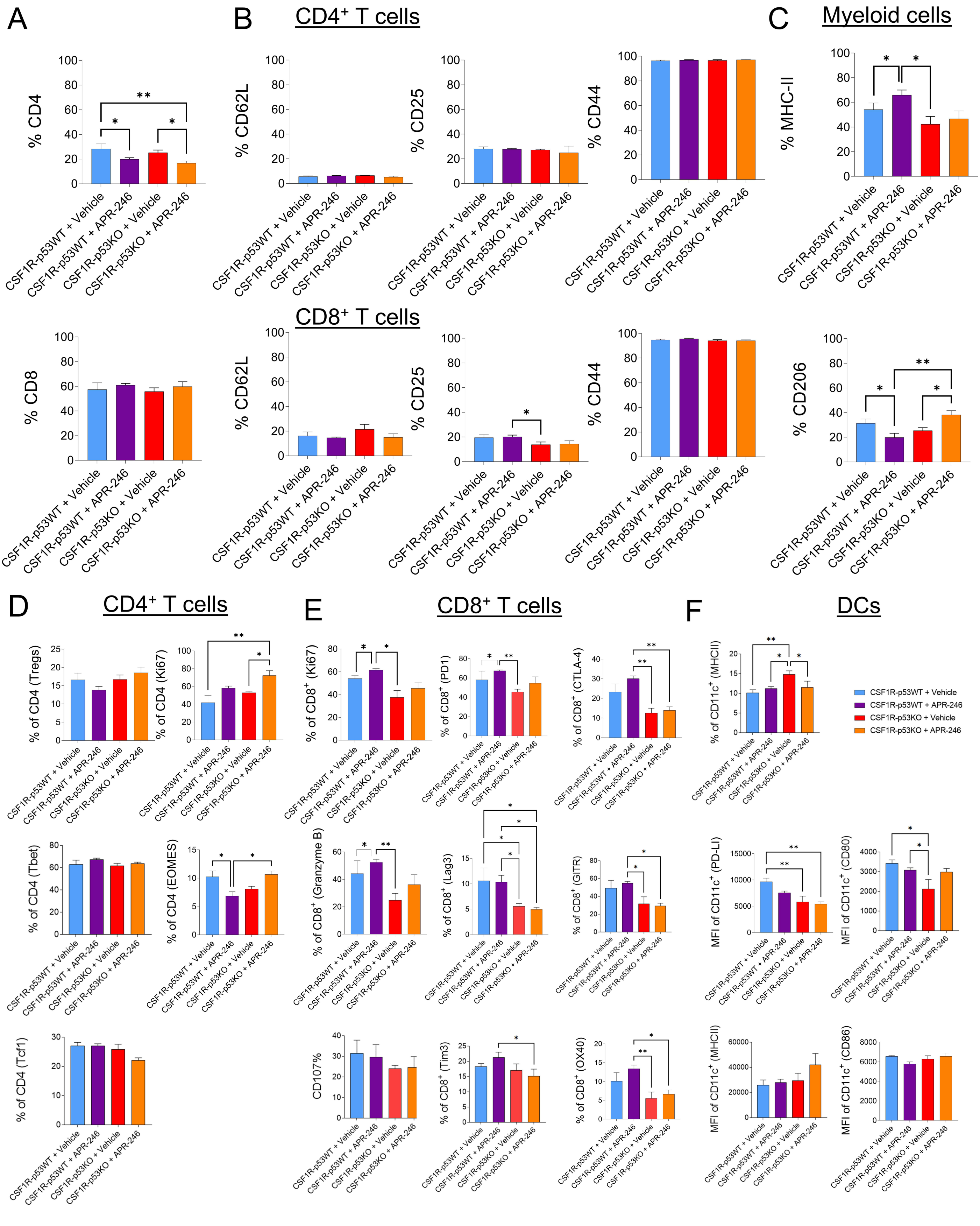

Supplemental Figure S4 (A-F) Multicolor flow cytometry analyses of live CD45+ gated cells of the TME. (N=5/group mean with SEM shown) (*P < 0.05; **P < 0.01; ***P < 0.001; ****P < 0.0001)

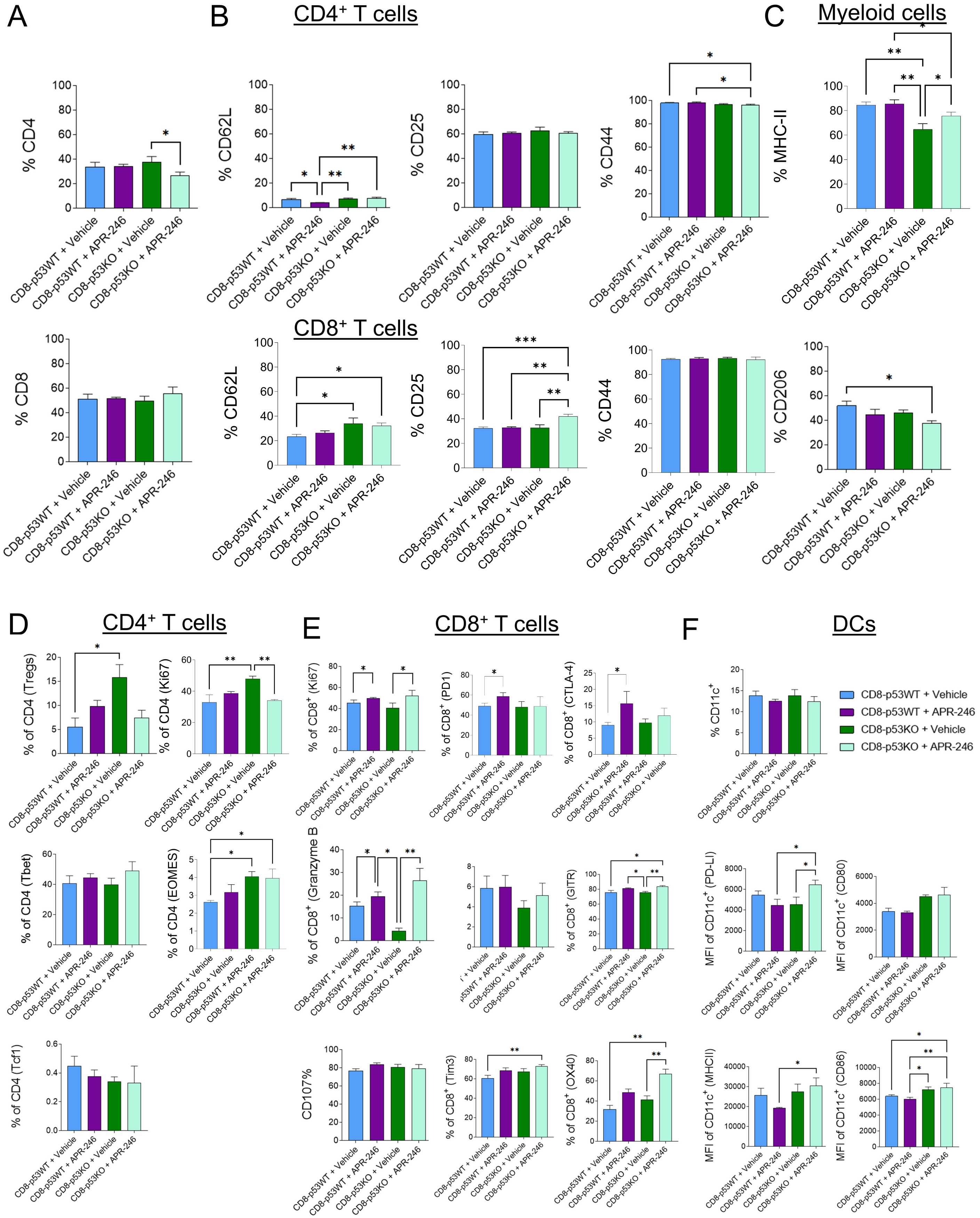

Supplemental Figure S5 (A-F) Multicolor flow cytometry analyses of live CD45+ gated cells of the TME. (N=4-5/group mean with SEM shown) (*P < 0.05; **P < 0.01; ***P < 0.001; ****P < 0.0001)

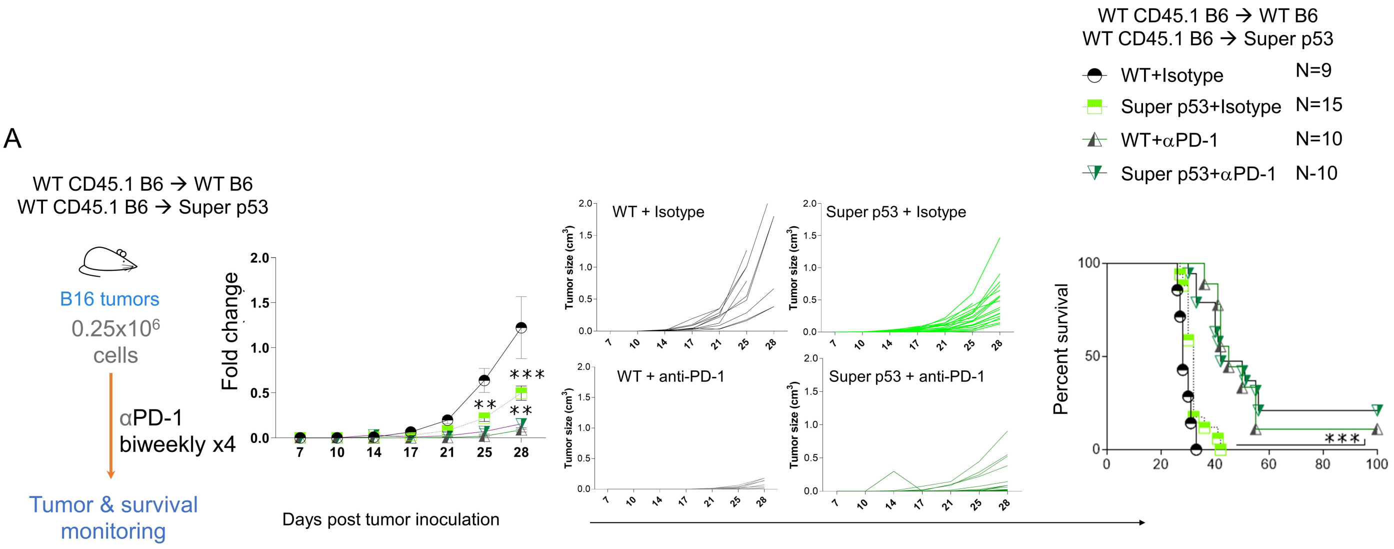

Supplemental Figure S6: Schematic of treatment groups, tumor growth curve and Kaplan Meier survival is depicted (Plots depicting two pooled experiments; N=depicted in legend) for Super p53 vs. WT mice WT mice reconstituted with CD45.1 B6 bone marrow, then inoculated with B16-melanoma tumors and treated with anti-PD-1 antibody. (*P < 0.05; **P < 0.01; ***P < 0.001; ****P < 0.0001)

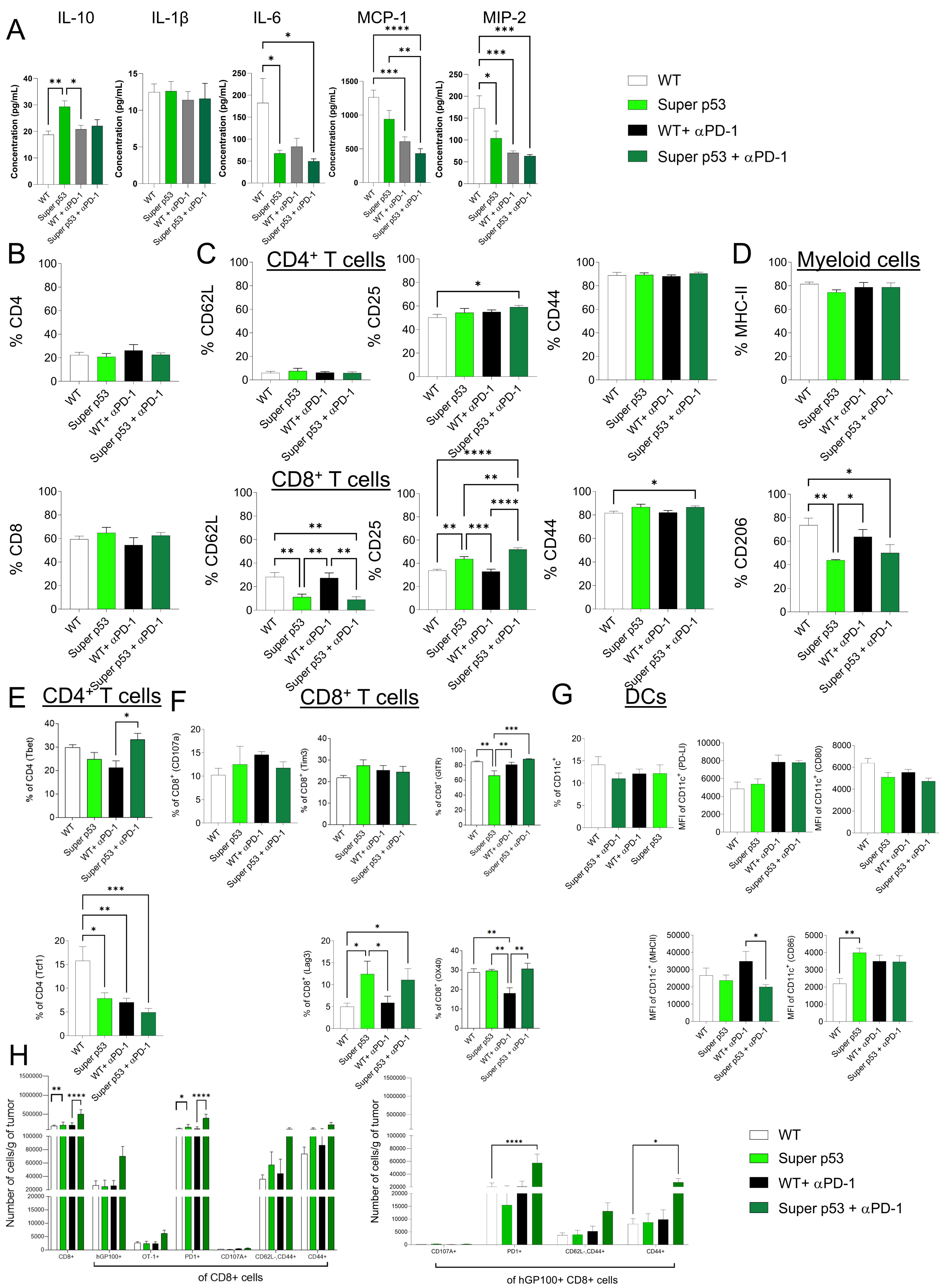

Supplemental Figure S7 (A) Murine cytokine array as performed on tumor lysates (N=4-5/group, performed in duplicates; mean with SEM shown) **(B-G)** Multicolor flow cytometry analyses of live CD45⁺ gated cells of the TME. (N=5/group mean with SEM shown) **(H)** Number of putative melanoma specific GP100⁺ TCR⁺ T cells (vs control SIINFEKL-specific OT-1 TCR⁺ T cells) (left panel) and their phenotype (right panel) (*P < 0.05; **P < 0.01; ***P < 0.001; ****P < 0.0001)

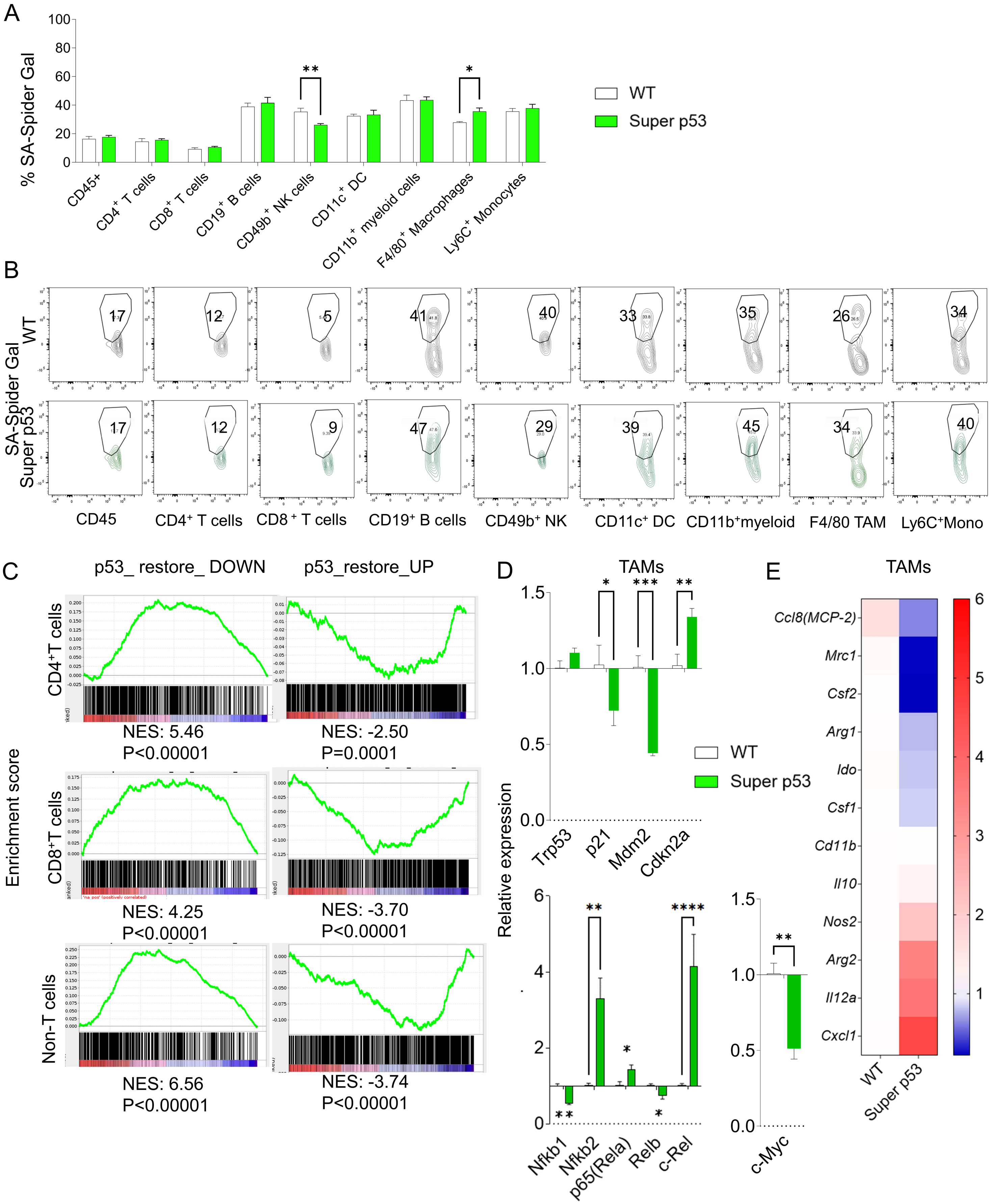

Supplemental Figure S8: (A) Senescence-associated beta-gal staining in different compartments of the TME in WT and Super p53 mice (N=5/group mean with SEM shown) **B)** Representative plots of senescence-associated beta-gal staining of each immune subtype in the TME **(C)** $CD45^+CD4^+$T cells, $CD45^+CD8^+$T cells, ($CD45^+CD4^-CD8^-$) non-T cells were sorted from TME of B16 tumors in wild type (WT) or super p53 mice, and RNA seq was performed. GSEA plot evaluating changes in the p53 pathway depending on p53 expression are depicted. (N=3/group) **(D-E)** $CD45^+TCRb^-CD11b^+F4/80^+$ TAMs were sorted at day 13 of tumor growth and quantitative RT-PCR was performed. (N=4/group, was performed in triplicates and mean with SEM shown)**(D)** Relative expression of genes related to p53 signaling, NF-kB and c-myc, and **(E)** Heatmap depicting expression of M1/M2 genes (*P < 0.05; **P < 0.01; ***P < 0.001; ****P < 0.0001)

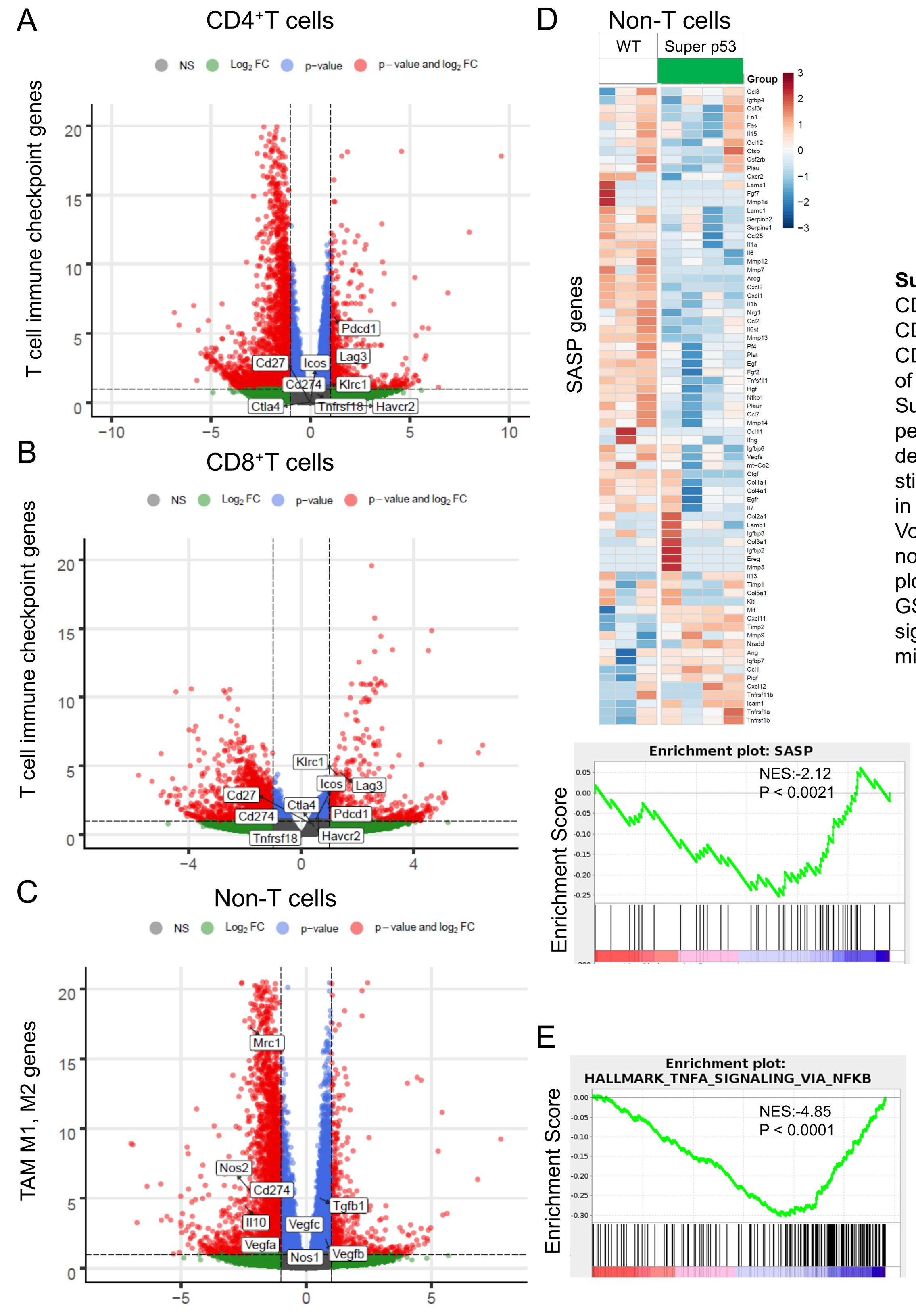

Supplemental Figure S9: : (A-E) CD45$^+$CD4$^+$ (CD4$^+$ T cells), CD45$^+$CD8$^+$(CD8$^+$ T cells), CD45$^+$CD4$^-$ CD8$^-$ (non-T) cells were sorted from TME of B16 tumors that had been implanted in Super p53 or WT mice, and RNA seq was performed. (N=3/group) Volcano plots depicting gene expression of select co-stimulatory and immune checkpoint genes in **(A)** CD4$^+$ and **(B)** CD8$^+$ T cells. **(C)** Volcano plots of select M1/M2 genes in non-T cells **(D)** Heatmap and enrichment plot of SASP gene set in non-T cells. **(E)** GSEA plot evaluating changes in NFkB signaling pathways in super p53 vs. WT mice.

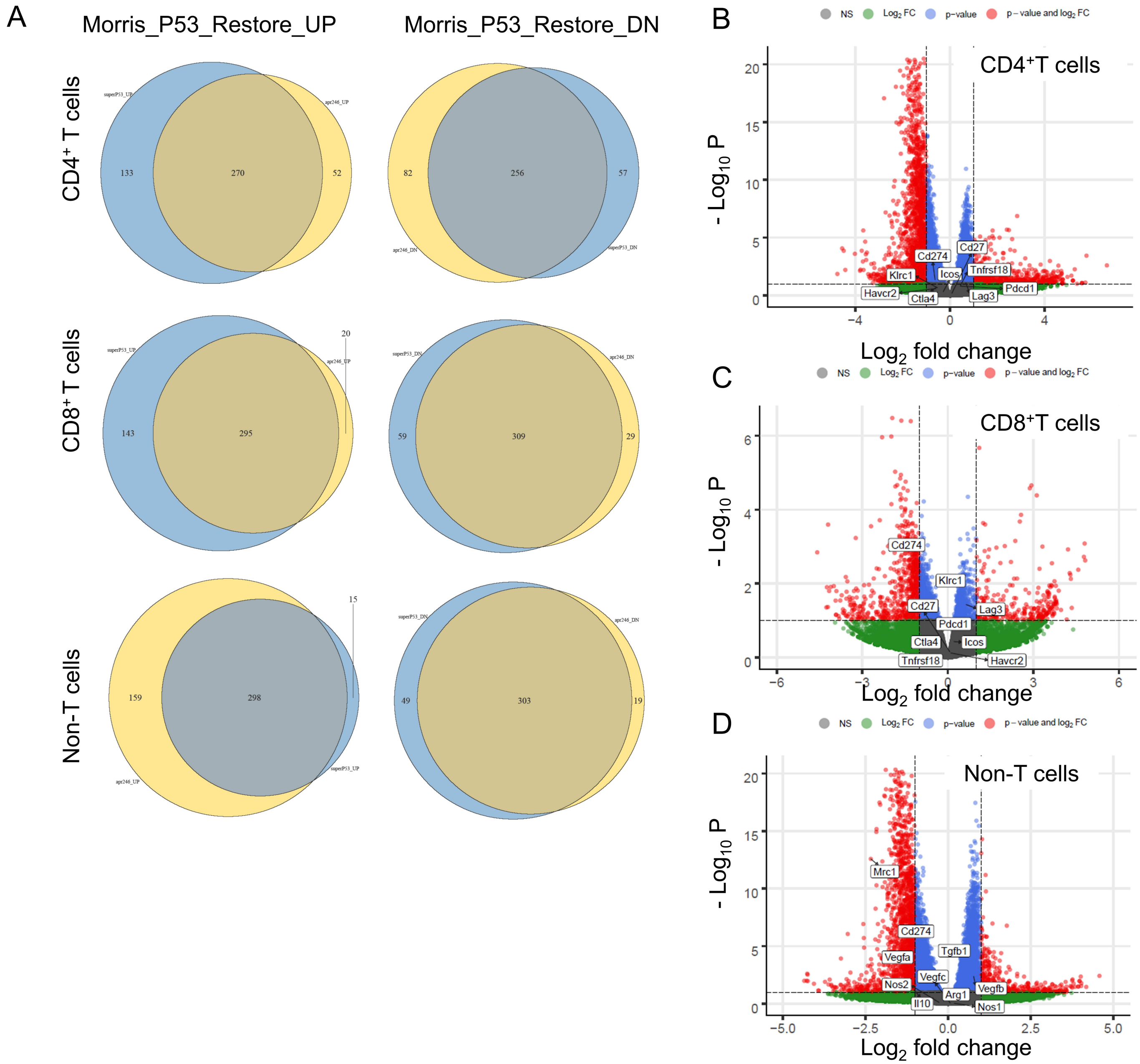

Supplemental Figure S10: (A-D) CD45$^+$CD4$^+$T cells (CD4$^+$T cells), CD45$^+$CD8$^+$T cells (CD8$^+$T cells), CD45$^+$CD4$^-$CD8$^-$ (non-T) cells were sorted from TME of B16 tumors inoculated in wildtype mice, and then treated with APR-246 or vehicle (control) and RNA seq performed.(N=4/group) **(A)** Venn diagrams depicting sets of core enrichment genes related to p53-pathways comparing cells from tumors of super p53 and APR-246-treated mice. (**(B, C)** Volcano plots depicting gene expression of select co-stimulatory and immune checkpoint genes in **(B)** CD4$^+$ and **(C)** CD8$^+$ T cells. **(D)** Volcano plots of select M1/M2 genes in non-T cells

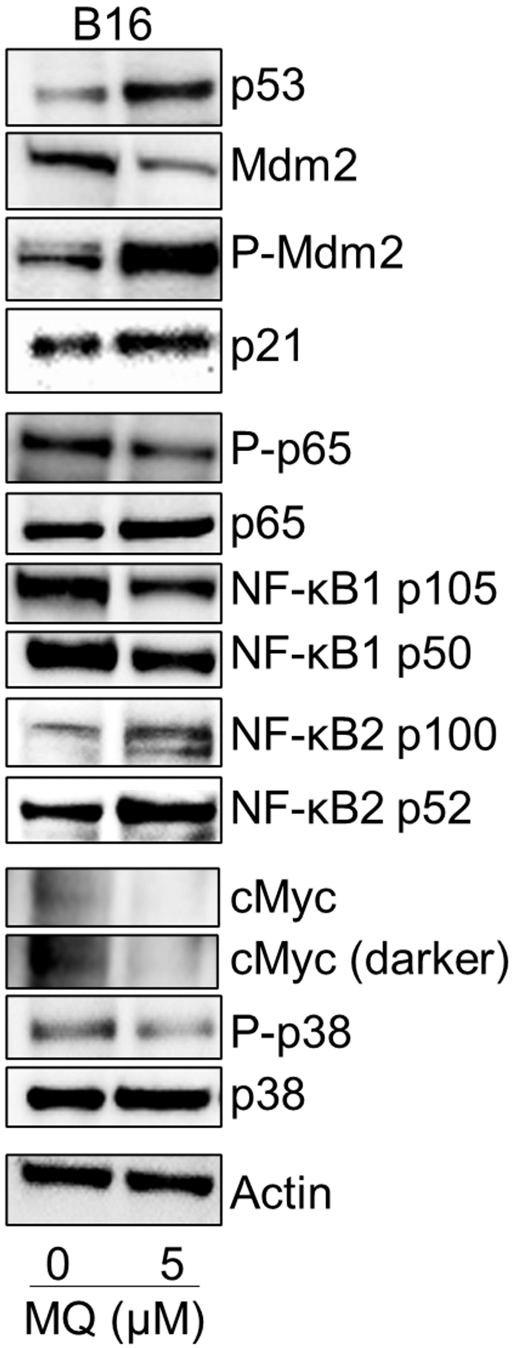

Supplemental Figure S11 (A) Western blot assessing the expression of key members of p53, NF-kB and MAPK pathways in B16 cells treated with MQ

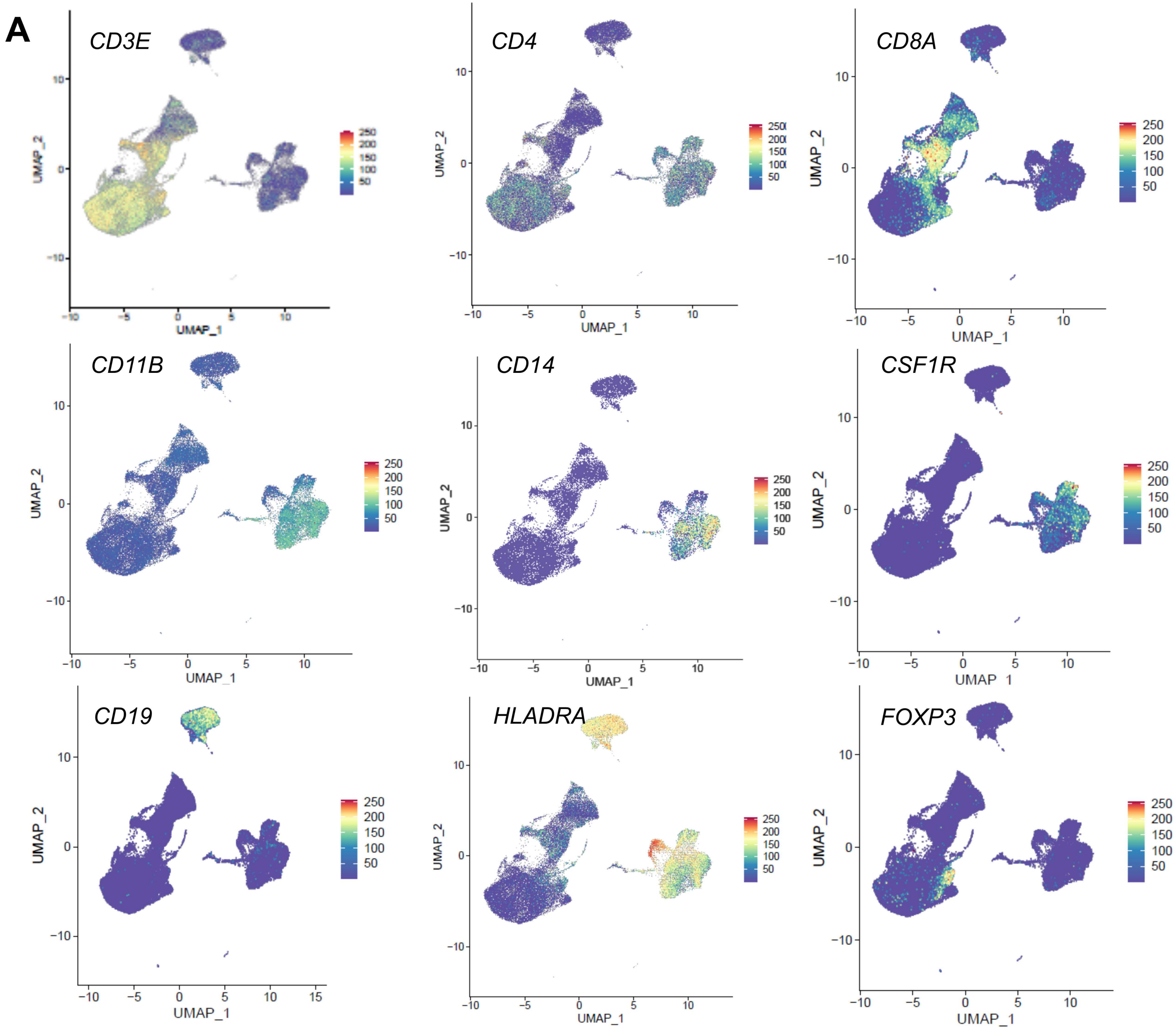

Supplemental Figure S12: Expression of lineage genes in the CiteSeq data depicted in Figure 6

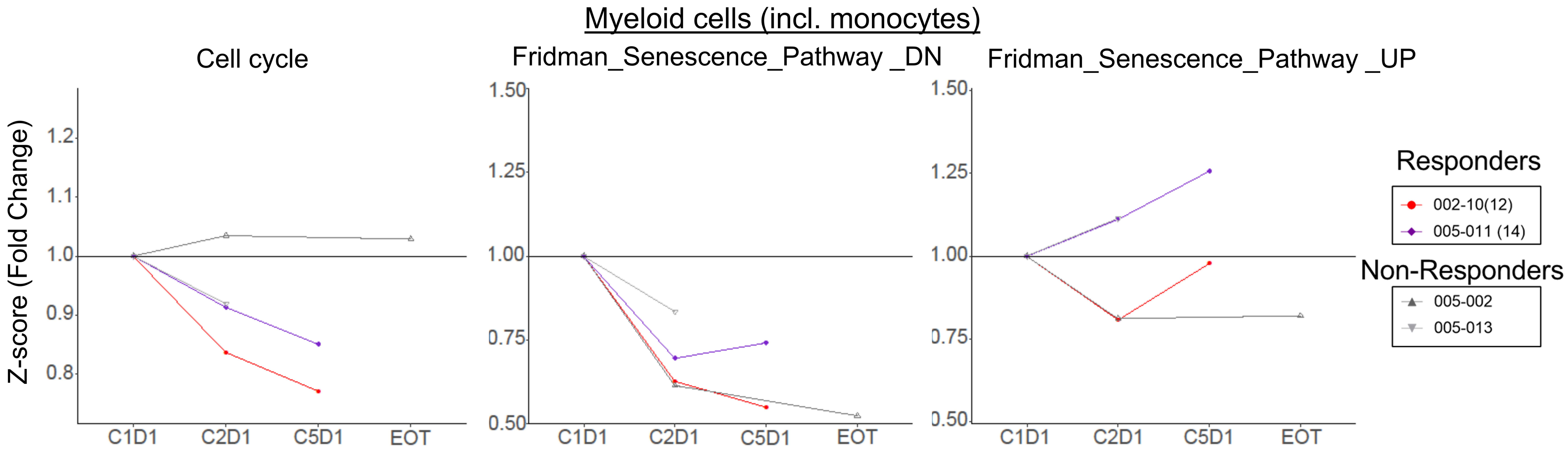

Supplemental Figure S13: PBMCs were collected prior to cycle 1 day 1 (C1D1), cycle 2 day 1 (C2D1), cycle 5 day 1 (C5D1) or at the end of treatment (EOT). Tumors of patients #002-10(12) and #005-11(14) displayed reduction in size while those from #005-02 and #005-13 continued to progress. GSEA analysis of myeloid cells including monocytes for cell cycle and senescence and fold change of Z-scores at different timepoints proportional to that at C1D1 are depicted.

Supplementary Tables

Table S1: Tumor responses from the patients shown.

Patient ID	Malignancy	Tumor response
002-010 (12)	Squamous Non small cell lung cancer (NSCLC)	Responder:<br>27% reduction in tumor size at 1st response assessment after 3 cycles of therapy<br>SD by RECIST, confirmed at 15 and 21 weeks
005-011 (14)	Squamous Non small cell lung cancer (NSCLC)	Responder:<br>11% reduction in tumor size at 1st response assessment after 3 cycles<br>SD by RECIST, confirmed at 15 and 21 weeks
005-002	Squamous Non small cell lung cancer (NSCLC)	Non-responder
005-013	Squamous Non small cell lung cancer (NSCLC)	Non-responder

Table S2: Mouse antibodies for flow cytometry

Antigen	Fluorophore	Clone	Company	Catalog #
CD62L	BUV395	MEL-14	BD	740218
CD107a	PE	1D4B	BD	558661
CD45	BUV563	30-F11	BD	612924
CD8	BUV615	53-6.7	BD	613004
CD49b	BUV661	HMα2	BD	741523
CD11c	BUV737	HL3	BD	612796
CD4	BUV805	RM4-4	BD	741913
Tbet	BV421	4B10	Biolegend	644816
p53	AF647	1C12	Cell Signaling	2533S
PD-L1	BV480	MIH5	BD	746275
F4/80	Pac Orange	BM8	Invitrogen	MF48030
CD11b	BV510	M1/70	Biolegend	101263
Ly6C	BV570	HK1.4	BioLegend	128029
Tim-3	SB600	8B.2C12	Invitrogen	63-5871-82
CD19	SB645	eBio1D3	Fisher	64-0193-82
CD86	SB702	GL1	Invitrogen	67-0862-82
GITR	BV750	DTA-1	BD	747402
PD-1	BV785	29F.1A12	BioLegend	135225
Lag3	BB515	C9B7W	BD	564672
CD80	AF488	16-10A1	BioLegend	104715
IA/IE	SparkBlue550	M5/114.15.2	Biolegend	107662
OX40	PE-Dazzle594	OX-86	BioLegend	119418
Eomes	PE-Cy5	Dan11mag	Invitrogen	15-4875-82
Ly6G	PerCP	1A8	BioLegend	127654
CD206	PerCP-Cy5.5	C068C2	BioLegend	141715
FoxP3	PerCP-eF710	FJK-16s	Invitrogen	46-5773-82
CTLA4	PE-CY7	UC10-4B9	Invitrogen	25-1522-82
Granzyme B	APC	GB11	Biolegend	372203
Tcf1	AF647	S33-966	BD	566693
CD25	Spark NIR685	PC61.5	Biolegend	102070
CD44	AF700	IM7	Invitrogen	56-0441-82
Ki67	APC-eF780	SolA15	Invitrogen	47-5698-82
CD3	APC-Fire810	17A2	Biolegend	100267

Supplementary Information

Conflict of interest: AG has consulted for Adivo Associates LLC, Group H and CPRIT. JM is a consultant for GlaxoSmithKline France.TM and JDW are coinventors on patent applications related to CD40 and in situ vaccination (PCT/US2016/045970), filed by MSKCC. JDW is a consultant for Amgen, Astellas, AstraZeneca, Bayer, Bicara Therapeutics, Boehringer Ingelheim, Bristol Myers Squibb, Chugai, Daiichi Sankyo, Dragonfly, Eli Lilly, F Star, Georgiamune, Idera, Imvaq Therapeutics, Kyowa Kirin, Merck, Psioxus, Recepta, Sellas, Surface Oncology, Syndax, Syntalogic Pharmaceuticals, Truvax, Trishula, and Werewolf Therapeutics. JDW receives grant/research support from Bristol Myers Squibb and Sephora. JDW is a consultant and has equity in Tizona Pharmaceuticals, Imvaq Therapeutics, BeiGene, Linneaus Therapeutics, Apricity, Arsenal IO, Georgiamune, Trieza, Maverick Therapeutics, and Ascentage Pharma and is an inventor on patents: Xenogeneic (Canine) DNA Vaccines (US 7,556,805), alphavirus replicon particles expressing TRP2 (PCT/US2010/030423), myeloid-derived suppressor cell (MDSC) assay (PCT/US2013/027475), Newcastle disease virus for cancer therapy (US 10,251,922), vaccinia virus mutants useful for cancer immunotherapy (with TM, US 16/612,127), anti-CD40 agonist mAb fused to monophosphoryl lipid A (MPL) for cancer therapy (with TM, US 15/750,496), antigenbinding proteins targeting melanoma differentiation antigens and uses thereof (US 16/388,245) anti–PD-1 antibody (with TM, US 10,323,091), anti-CTLA4 antibodies (with TM, US 10,144,779), and anti-GITR antibodies and methods of use thereof (with TM, US 10,155,818; US 10,280,226). TM is a consultant for ImmunOs Therapeutics and Pfizer; is cofounder of and equity holder in Imvaq Therapeutics; receives research funding from Bristol Myers Squibb, Surface

Oncology, Ikena Oncology, Infinity Pharmaceuticals, Peregrine Pharmaceuticals, Adaptive Biotechnologies, Leap Therapeutics, and Aprea Therapeutics; and is an inventor on patent applications related to oncolytic viral therapy (US 16/980,282), alpha virus–based vaccine (with JDW, PCT/US2010/030423), neo antigen modeling (with JDW, US 16/478,818), and OX40 (US 62/262,371). SB have received royalties from Agenus. TM are coinventors on patent applications related to OX40 antibodies (PCT/US2016/064794), filed by MSKCC. DH is an inventor on patent applications related to contraceptive vaccines (PCT/US2004/006216 and PCT/US2003/029257). R.Z. is inventor on patent applications related to work on GITR, PD-1, and CTLA-4. R.Z. is consultant for Leap Therapeutics and iTEOS Belgium SA. S.W.L. is a founder and member of the scientific advisory board of Blueprint Medicines, Mirimus, ORIC Pharmaceuticals and Faeth Therapeutics, and is on the scientific advisory board of Constellation Pharmaceuticals and PMV Pharmaceuticals.
